# Multimodal Sensing and Modeling of Endocrine Therapy Adherence in Breast Cancer Survivors

**DOI:** 10.1145/3770864

**Published:** 2025-12-02

**Authors:** FANGXU YUAN, NAVREET KAUR, ZHIYUAN WANG, MANUEL GONZALES, CRISTIAN GARCIA ALCARAZ, GABRIEL ESTRELLA, KRISTEN J. WELLS, LAURA E. BARNES

**Affiliations:** Department of Systems and Information Engineering, University of Virginia, USA; Department of Systems and Information Engineering, University of Virginia, USA; Department of Systems and Information Engineering, University of Virginia, USA; SDSU/UC San Diego Joint Doctoral Program in Clinical Psychology, San Diego State University, USA; SDSU/UC San Diego Joint Doctoral Program in Clinical Psychology, San Diego State University, USA; Department of Educational Psychology, San Diego State University, USA; Department of Psychology, San Diego State University, USA; Department of Systems and Information Engineering, University of Virginia, USA

**Keywords:** medication adherence, breast cancer survivors, breast neoplasm, multimodal data, temporal modeling, machine learning, digital health

## Abstract

Many breast cancer survivors are prescribed daily oral medications called endocrine therapy that prevent cancer recurrence. Despite its clinical importance, maintaining consistent daily adherence remains challenging due to the dynamic and interrelated influences of behavioral, physiological, and psychological factors. While prior studies have explored adherence prediction using mobile sensing, they often rely on single-modality data, limited temporal granularity, or aggregate-level modeling—limiting their ability to capture short and long-term behavioral variability and to facilitate deeper understanding of non-adherence and tailored interventions. To address these gaps, we propose a multimodal sensing framework that explicitly models daily adherence dynamics using temporally adaptive inputs. We recruited a sample of breast cancer survivors (*N* = 20) and collected longitudinal data streams including wearable-derived physiological features (Fitbit), medication event monitoring system (MEMS) data, and ecological momentary assessments (EMAs). Using multimodal data across varying time windows, we examined whether recent patterns in behavioral, physiological, psychological, and environmental factors improve the prediction of next-day endocrine therapy adherence. Our results demonstrate the feasibility of using multimodal sensing data to predict daily adherence with moderate accuracy. Moreover, models integrating multimodal data consistently outperformed those relying on a single modality. Importantly, we observed that the predictive value of each modality varied depending on the temporal proximity of the input signals, underscoring the importance of modeling immediate and longer-term behavioral patterns. The findings offer valuable insights for advancing adherence monitoring systems, suggesting that incorporating personalized and temporally adaptive data fusion strategies may significantly enhance the effectiveness of intervention design and delivery.

## 1 INTRODUCTION

Breast cancer survivors with estrogen and/or progesterone receptor positive tumors are typically prescribed adjuvant endocrine therapy, such as Tamoxifen or aromatase inhibitors, for 5 to 10 years to prevent recurrence [15]. However, maintaining adequate adherence to these long-term therapies poses significant challenges. Adherence rates reported in the literature vary considerably, ranging from about 41–88% for tamoxifen and 50–91% for aromatase inhibitors [34]. Documented barriers to adherence include the prolonged duration of treatment, burdensome side effects (e.g., hot flashes, joint pain, fatigue), and psychosocial factors such as low perceived benefit and limited social support [8]. Adherence is especially vulnerable during the transition from active treatment to survivorship care. This period requires survivors to integrate long-term medication-taking into their daily routines while managing lingering side effects and re-engaging with normal life activities [23]. Given the extended nature of endocrine therapy and the reduced clinical oversight during survivorship, scalable solutions that offer ongoing support and monitoring of adherence are urgently needed.

Given the complex and multifactorial nature of medication adherence, theory-driven models are essential for capturing the underlying behavioral mechanisms. Research grounded in Social Cognitive Theory (SCT) [3] emphasizes that health behaviors are shaped by the dynamic interplay of personal factors (e.g., self-efficacy, perceived barriers), behavioral patterns (e.g., routines, adherence history), and environmental influences (e.g., social support, healthcare system interactions) [4]. According to SCT, enhancing individuals’ self-efficacy and providing supportive environmental cues can strengthen health-related decision-making and improve adherence behaviors [5]. These theoretical insights underscore the need to examine how multiple influences operate together over time to affect treatment engagement. Doing so requires comprehensive, longitudinal data that capture both explicit determinants of adherence (e.g., self-reported barriers) and implicit indicators (e.g., daily routines, physical activity, sleep quality), enabling a more nuanced understanding of how personal, behavioral, and contextual factors jointly shape adherence patterns.

Recent advances in passive sensing technologies—including smartphones, wearable devices, and smart pill bottles—offer unprecedented opportunities to continuously and unobtrusively monitor longitudinal behavioral patterns relevant to medication adherence. These technologies enable the real-time observation of daily activities, sleep patterns, social interactions, and medication-taking behaviors, providing a holistic view of the factors influencing adherence [17, 32]. This scalable and non-intrusive approach allows healthcare providers and researchers to understand nuanced behavioral trends and barriers, ultimately facilitating more personalized and timely intervention strategies.

Despite ongoing progress, significant gaps remain in the literature. First, while many prior studies rely on unimodal or single-device sensing approaches [47], few have attempted to holistically integrate behavioral, physiological, and psychological data into a unified adherence modeling framework. Second, even among multimodal approaches, there is a tendency to analyze data streams in isolation, neglecting the temporal interdependencies that may influence adherence behavior. Third, strategies for optimizing multimodal data fusion and for quantifying the individual contribution of each modality to prediction accuracy have received relatively little attention.

### 1.1 Research Questions

To address the complex and multifactorial nature of adjuvant endocrine therapy medication adherence among breast cancer survivors, we posed the following research questions:

1. **RQ1:** To what extent can multimodal data representing distinct Social Cognitive Theory constructs—personal, environmental, and behavioral—accurately predict daily medication adherence?
2. **RQ2:** How does predictive performance vary between unimodal and multimodal models, and what does this reveal about the complementary value of different data sources?
3. **RQ3:** How do behavioral and psychological signals contribute to adherence prediction at varying temporal proximities (e.g., immediate vs. lagged effects)?

### 1.2 Modeling Framework

To answer these questions, we proposed a multimodal sensing and modeling framework grounded in SCT, which emphasizes the joint influence of personal, behavioral, and environmental factors on health behaviors. Our approach integrated longitudinal data from wearable devices (Fitbit), ecological momentary assessments (EMAs), and medication event monitoring system (MEMS) devices. This multimodal strategy enables continuous, real-world monitoring of adherence-related behaviors, physiological states, and psychological factors among a cohort of 20 early-stage breast cancer survivors over a six-month period.

We implemented a two-tier predictive modeling pipeline that first trained modality-specific Long Short-Term Memory (LSTM) networks and then fused their outputs using an optimized soft-voting ensemble. To ensure robust generalizability, we adopted a participant-independent leave-one-participant-out (LOPO) cross-validation strategy. Additionally, we conducted modality ablation analyses to evaluate the individual and combined predictive value of each sensing stream.

### 1.3 Contributions

This work addresses critical gaps in the existing literature on medication adherence modeling, which often relies on single-modality data, lacks temporal granularity, or fails to capture dynamic, personalized adherence patterns. In contrast, our approach leverages temporally adaptive, multimodal inputs to improve the fidelity and personalization of adherence prediction. Specifically, we make the following key contributions:

1. We introduce a unified multimodal sensing framework that integrates longitudinal data from Fitbit, MEMS, and EMA, enabling fine-grained modeling of daily medication adherence behavior.
2. We develop a two-tier predictive pipeline that combines modality-specific sequence-based deep learning models with an optimized soft-voting fusion strategy, preserving modality-specific temporal dynamics while enhancing overall predictive performance.
3. We demonstrate that multimodal integration significantly outperforms unimodal models and that each modality contributes variably depending on its temporal proximity to the adherence event, validating the necessity of adaptive temporal modeling.
4. We provide a detailed modality-wise performance analysis, offering practical design insights for future adherence monitoring systems that aim to be both scalable and personalized, particularly in cancer survivorship contexts.

## 2 RELATED WORK

To contextualize our contribution, we begin by discussing work at the intersection of Social Cognitive Theory (SCT) and health. We then review research on mobile and wearable sensing for modeling psychological and behavioral health outcomes, particularly within the domains of mental health and oncology. This body of work demonstrates how multimodal data—including physiological, behavioral, contextual, and self-reported signals—can be leveraged to model affective states, detect psychological risk, and support personalized, real-time health interventions. Finally, we turn to techniques aimed at predicting medication adherence and detecting individual pill ingestion events.

### 2.1 Social Cognitive Theory-based Behavior Modeling

Social Cognitive Theory (SCT) provides a widely adopted framework for understanding health behavior change through the interaction of personal, behavioral, and environmental factors [28]. Islam et al. [24] reviewed SCT-based health interventions and emphasized the central role of self-efficacy and observational learning across diverse populations and delivery modalities. In the context of cancer survivorship, Kaur et al. [27] developed theory-guided models that integrate both more static measures (e.g., trait-level characteristics or cancer history) and dynamic data (e.g., MEMS-based adherence) to identify patients at risk for non-adherence, highlighting the value of multimodal and temporally resolved predictors. Similarly, Baglione et al. [2] proposed the Multiscale Modeling and Intervention (MMI) system—a sensor-based framework that captures *in situ* behavioral, environmental, and personal contexts to enable real-time, personalized adherence interventions grounded in SCT.

Beyond oncology, SCT principles have been operationalized in mHealth interventions targeting other chronic conditions. Sze et al. [46] introduced a step-tracking intervention that improved both physical activity and self-efficacy among individuals with type 2 diabetes. Hartch et al. [22] evaluated a smartphone-based medication adherence app that enhanced self-efficacy and treatment engagement in underserved populations with chronic illness.

While these efforts underscore the utility of SCT in guiding intervention design, many rely heavily on self-reported data and fall short in capturing the dynamic interactions among personal, environmental, and behavioral factors necessary to support adaptation in real-world contexts. To address these limitations, recent work—including the present study-explores the integration of active and passive sensing with theory-driven models to enable real-time, personalized adherence support.

### 2.2 Multimodal Mobile and Wearable Sensing for Health

Mobile sensing has emerged as a powerful method for continuously capturing psychological and behavioral states in naturalistic settings. Larrazabal et al. [31] demonstrated that wearable-derived biobehavioral, contextual, and trait-level data can effectively predict momentary fluctuations in state social anxiety during virtual social interactions, highlighting the importance of social context and multimodal integration in mental health modeling.

Beyond anxiety, recent work has extended passive sensing to more complex mental health conditions such as depression. Lamichhane et al. [30] used smartphone and wearable data to assess depression severity, showing that passive features can reliably reflect mental health status across diverse populations. Similarly, Saeb et al. [41] and Canzian and Musolesi [9] found that smartphone-derived mobility patterns correlate with depressive symptoms, underscoring the potential of location and movement data as passive indicators of mental health.

Building on this foundation, Islam et al. [25] introduced *FacePsy*, a system that leverages facial expressions and head gestures for naturalistic depression detection. Zhang et al. [55] combined smartphone-based sensing with large language models to predict affective states, demonstrating how advanced machine learning can enhance emotion recognition. In parallel, Shu et al. [44] employed wrist-worn heart rate and motion sensors to detect affective states in real-time, enabling applications in emotion-aware computing and real-world mood tracking.

In addition to emotion modeling, researchers have explored wrist-worn sensors for detecting medication-taking gestures. Fozoonmayeh et al. [16] developed a scalable smartwatch-based system that uses accelerometer and gyroscope data to monitor medication intake, supported by a cloud-based infrastructure for distributed data processing. Their classification models—including gradient boosted trees and random forests—achieved F1 scores above 0.97. Building on this, Odhiambo et al. [37] demonstrated that personalized neural networks can detect both protocol-guided and naturalistic pill-taking gestures with over 95% accuracy. These efforts highlight the feasibility of unobtrusive behavior-specific sensing for adherence monitoring and point toward future integration with real-time interventions such as reminder notifications or conversational agents.

While these studies illustrate the potential of passive sensing for health applications, there remains a relative paucity of research applying multimodal sensing frameworks specifically to medication adherence. Moreover, much of the existing work relies predominantly on smartphone-based sensors, limiting integration of richer physiological data streams [30, 41]. In contrast, our approach employs a multimodal sensing framework that integrates Fitbit-derived activity, sleep, and heart rate data with MEMS-based adherence monitoring and ecological momentary assessments (EMAs) of psychological, social, and environmental factors. This fusion enables continuous, fine-grained modeling of daily adherence behavior and supports the development of timely, personalized interventions.

In oncology, Valero-Ramon et al. [48] demonstrated the utility of integrating wearable-derived activity and sleep data with self-reported psychological outcomes—such as vulnerability, anxiety, and depression—to model time-varying risk among older adult cancer survivors. Using interactive process mining on real-world data, they identified behavioral patterns (e.g., prolonged sedentary behavior, irregular sleep) strongly associated with elevated emotional burden. Although their work focused on psychological risk rather than adherence, it underscores the value of combining multimodal sensing with frequent self-reports to uncover dynamic behavioral profiles—offering a foundation for personalized, context-aware interventions in cancer care.

The broader clinical potential of wearables in oncology has also been emphasized in recent systematic reviews [11], which highlight their utility in monitoring patient symptoms, treatment side effects, and quality of life. However, these reviews note that most existing studies have prioritized feasibility and acceptability, with relatively few advancing modeling frameworks capable of real-time behavioral prediction.

Wu et al. [50] introduced *CardioAI*, a prototype system that integrates wearable-derived physiological signals with a large language model-based voice interface to capture patient-reported symptoms and generate explainable cardiotoxicity risk scores. The system exemplifies the integration of passive and active sensing within an AI-assisted remote monitoring pipeline tailored to post-treatment cancer care. While *CardioAI* targets short-term cardiotoxicity risk and clinical workflow integration, our work extends multimodal sensing to model long-term behavioral adherence trajectories and daily symptom burden in cancer survivors. As described earlier, we combine wearable data (Fitbit), objective medication adherence monitoring (MEMS), and ecological momentary assessments (EMA) to enable fine-grained, temporally-aware modeling in support of personalized interventions.

### 2.3 Modeling and Detecting Medication Adherence with Mobile Sensing

Adherence to medication is influenced by a complex interplay of physiological, behavioral, social, environmental, and psychological factors. Research using mobile sensing to study medication adherence can be broadly categorized into three areas: (1) detecting medication-taking gestures from sensor data, (2) predicting adherence using contextual and longitudinal features, and (3) designing and evaluating integrated, technology-enabled adherence interventions.

1. *Sensor-based Detection of Medication-taking Activities.* An increasing number of studies leverage motion sensor data and smart dispensers to detect medication-taking behaviors. Mamun et al. [33] introduced AIMI, a knowledge-guided system that combines smartphone inertial sensors with prior medication records to detect intake gestures, demonstrating high accuracy across varied conditions. Nzeyimana et al. [36] developed DoseMate, a wrist-worn system that uses transfer learning and data augmentation to identify pill-taking gestures with F1 scores exceeding 96%. Aldeer et al. [1] proposed PillSense, a smart pill bottle equipped with motion and weight sensors to confirm pill dispensing events, offering a low-power, unobtrusive monitoring solution. In parallel, electronic medication event monitors, such as RxCap^1^ and Wisepill^2^ connected dispensers, have been widely adopted in clinical and research settings, logging bottle opening events in real-time as a proxy for ingestion [27]. A recent randomized clinical trial by Graetz et al. [18] directly employed the Wisepill device to monitor medication adherence among women prescribed adjuvant endocrine therapy. Participants used the device for one year alongside a mobile app for weekly symptom and adherence reporting. Although no significant improvement in adherence was observed across intervention arms, the integration of Wisepill with tailored feedback messages led to fewer high-cost healthcare encounters, suggesting that real-time adherence tracking, when coupled with symptom-triggered support, may offer ancillary benefits beyond medication-taking behavior. While these systems enable accurate detection of individual intake events, they are often siloed from broader behavioral or contextual models of adherence and do not necessarily contribute to understanding complex adherence patterns.
2. *Prediction of Adherence Using Contextual Features.* Beyond gesture recognition, other studies have modeled adherence behavior using contextual, temporal, and psychosocial features. Gu et al. [20] applied ensemble learning and LSTM-based time series models to self-injection adherence logs, showing how temporal dynamics can reveal behavioral patterns. However, self-injection involves complex preparatory steps and may not generalize to more routine oral medication regimens. In addition, many of these approaches rely on single-modality data and prioritize population-level prediction performance, often overlooking personalization and short-term behavioral variability.
3. *Integrated Sensing and Intervention Systems for Medication Adherence.* Other systems have adopted more holistic approaches to sensing and intervention. For instance, the SAMSON system [13] integrates mobile sensing and digital tools to support adherence and self-management among oncology patients. Baglione et al. [2] introduced the MMI platform, which combines smartphones, MEMS caps, wearable sensors, and beacons to contextualize adherence behaviors in breast cancer survivors. Similarly, Kaur et al. [27] proposed a longitudinal predictive model that incorporates less frequently changing psychosocial factors alongside dynamic adherence histories. Prior work has also demonstrated the potential of theory-driven modeling approaches: Kaur et al. [26] employed a randomized neural network framework guided by social cognitive theory and random utility theory to decode individual-level medication-taking behavior using MEMS-based adherence data and periodic survey responses. However, these prior efforts are either conceptual frameworks or lack the joint integration of high-frequency behavioral and physiological data—such as EMA and wearable data streams—potentially limiting their capacity to capture the dynamic, proximal signals that influence daily adherence behavior.

In contrast, our work uses a multimodal sensing framework that captures a holistic view of the context surrounding medication-taking. We combine MEMS-based adherence records, wearable Fitbit data, EMAs, and baseline surveys capturing socio-demographics and self-beliefs. MEMS and Fitbit provide passive, continuous monitoring, while EMA contributes proximal psychological and behavioral context. To integrate these heterogeneous sources, we propose a two-tier modeling pipeline that uses modality-specific LSTM models and an optimized soft-voting fusion strategy. To ensure fairness and generalizability, we evaluate model performance using macro-averaged balanced accuracy and F1 scores across participants, allowing participant-independent, fine-grained, and theory-driven adherence modeling.

## 3 POPULATION, DATA COLLECTION, AND PROCESSING

### 3.1 Population

We employed purposeful sampling to recruit 20 breast cancer survivors for a six-month longitudinal study. Recruitment occurred between January and October 2023 via ResearchMatch and targeted social media posts (e.g., Facebook). Interested individuals were directed to an online self-report eligibility screener hosted on Qualtrics, which assessed the following inclusion criteria:

1. English fluency (reading and speaking);
2. Age 21–70 years;
3. Diagnosis of stage 0-III breast cancer within the past five years;
4. Completion of primary treatments (surgery, radiation, chemotherapy), excluding ongoing endocrine therapy;
5. Current prescription of endocrine therapy medication for breast cancer recurrence prevention;
6. No physical impairments that would preclude use of the medication monitoring system;
7. Capacity to provide informed consent;
8. Willingness and ability to use the monitoring system for six months; and
9. No planned relocation during the study period.

Participants who met initial criteria completed a brief Zoom screening interview to confirm eligibility, followed by a 60–90 minute baseline assessment conducted remotely. All study procedures were approved by the San Diego State University (SDSU) Institutional Review Board (Protocol Number: HS-2022-0089-SMT). Table 1 summarizes selected demographic characteristics of the 20 participants.

During the study period, one participant was lost to follow-up and did not complete the study. Another participant did not have any data from the RxCap device due to device malfunctioning, rendering their data unusable. Hence, both of these participants had to be removed from the analysis. Additionally, one participant completed study visits but did not provide any EMA survey data, resulting in partial missingness for analyses involving EMA-derived features.

### 3.2 Outcome Variable

We operationalized daily medication adherence using data from the RxCap MEMS, consistent with established practices in chronic oral therapy studies [38, 54].

*Daily Adherence Definition.* Daily adherence was determined based on the interval between consecutive bottle-opening events. A day was classified as adherent if the time elapsed since the previous dose fell within a clinically acceptable window of 18 to 30 hours (24 ± 6 hours). This threshold captures acceptable variability in dosing behavior while maintaining therapeutic consistency:

$$\text{Daily}\;\text{adherence}:\;\text{Inter-dose}\;\text{interval}\in \left[18\;\text{h},\;30\;\text{h}\right]$$

*Evaluation Metrics.* To assess model performance in predicting daily adherence, we employed two complementary metrics that account for class imbalance and ensure fair evaluation across both adherence and non-adherence outcomes.

- **Macro-Averaged Balanced Accuracy (Macro BA):** Balanced Accuracy is defined as the average of sensitivity (recall) for the positive class and specificity for the negative class. The macro-averaged version computes this metric independently for each class and then averages the results, assigning equal weight to adherent and non-adherent days.
- **Macro-Averaged F1-Score (Macro F1):** The F1-score is the harmonic mean of precision and recall. The macro-averaged version calculates the F1-score for each class separately and then averages them, ensuring that both classes contribute equally to the overall performance score.

Medication adherence datasets often exhibit class imbalance, with adherent days substantially outnumbering non-adherent days. In such settings, standard accuracy can be misleading, as it tends to reflect the performance on the majority class. By contrast, both Macro BA and Macro F1 provide a more balanced evaluation by giving equal importance to each class, ensuring that model performance on the minority non-adherent class is not overshadowed by the majority.

### 3.3 Multimodal Input Data

We integrated four complementary data streams to capture both subjective and objective facets of participants’ daily lives:

*Ecological Momentary Assessments (EMAs)*. Participants responded to brief prompts up to five times per day via a custom smartphone app built on the Sensus framework [51], providing real-time self-reports of mood, stress, medication side effects, and context of medication-taking behaviors (e.g., location, social interactions) [42].

*Wearable Data*. Each participant wore a Fitbit Sense smartwatch continuously, yielding minute-level data across multiple domains, including physical activity (e.g., steps, active minutes, sedentary bouts), sleep (e.g., total sleep time, sleep stages), and cardiovascular function (e.g., resting heart rate, heart rate variability).

*Medication Event Monitoring System (MEMS)*. RxCap medication bottles were equipped with MEMS, which time-stamped each opening to record the objective dosing behavior.

*Baseline Survey Data*. Structured surveys were administered after enrollment via Qualtrics, capturing demographic characteristics, clinical history, and psychosocial measures (e.g., self-efficacy, medication beliefs).

#### 3.3.1 EMA Data.

We deployed the Sensus mobile sensing framework to deliver EMAs on participants’ personal smartphones [51]. Surveys were administered across five schedules to balance data granularity and participant burden: daily morning, daily evening, random (every three days), biweekly, and monthly intervals.

*Morning EMA*. Triggered daily at 09:00 (available until 13:00).

*Evening EMA*. Triggered daily at 19:00 (available until 00:00).

*Random EMA*. Triggered every three days at 19:00, remaining open until completed or until the next random prompt.

*Biweekly EMA*. Triggered every fourteen days at 12:00 (available for seven days).

*Monthly EMA*. Triggered every twenty-eight days at 12:00 (available for seven days).

*Missing Data Imputation and Feature Propagation*. To construct a continuous daily dataset, we first generated a complete date grid spanning each participant’s EMA participation period and merged existing records into this panel, explicitly marking days without completed surveys. Item-level missingness was then addressed using a two-stage imputation strategy:

1. Categorical items: For each participant, missing categorical responses were imputed using the mode calculated over a seven-day rolling window preceding the missing value.
2. Numerical items: Missing numerical responses were imputed using multivariate imputation via scikit-learn’s IterativeImputer, leveraging correlations among EMA features.

To transform episodic survey responses into daily predictors, imputed values from random, biweekly, and monthly EMAs were forward-filled across their corresponding intervals (three, fourteen, and twenty-eight days, respectively), aligning all EMA-derived features on a daily basis with the morning and evening assessments. The complete EMA item batteries for each schedule are provided in Appendix A.

#### 3.3.2 Fitbit Data.

Participants received Fitbit devices to continuously record minute-level step counts, heart rate, and sleep data. We defined a *valid* day as one with ≥ 480 min (8 h) of wear time. Applying this criterion yielded 2,593 valid person-days from *n* = 18 participants (range: 50–195 days per participant). Across all valid days, the mean wear time was 1,272.6 min/day (min: 480 min; max: 1,440 min).

*Aggregated daily metrics*. Daily summaries were obtained via Fitabase, including physical activity (e.g., total steps, active/sedentary minutes), sleep (e.g., total sleep time, sleep efficiency), and heart rate (e.g., resting HR, HR variability).

*Derived minute-level features*. Raw minute-level data were processed [19] as follows: We computed daily behavioral measures of activity intensity (e.g., step variability, number of active bouts) and heart-rate dynamics, including first-order statistics (e.g., skewness, kurtosis) and second-order texture features (e.g., local homogeneity), to capture temporal variability and complexity.

*Missing data imputation*. Missing Fitbit data were imputed separately for each participant. First, to preserve local temporal patterns, partial missingness within time series was addressed using linear interpolation based on adjacent days. Subsequently, remaining missing values were imputed using an Iterative Imputer employing a gradient-boosting regression model, capturing multivariate relationships among daily Fitbit features. Variables with more than 30% missing values after imputation were excluded, resulting in a final set of 57 predictors (see Appendix B)

#### 3.3.3 Demographics and Baseline Survey Data.

Participants completed structured baseline assessments after enrollment via Qualtrics. Baseline measures captured a broad range of psychological, behavioral, and clinical factors, including health-related quality of life, comorbidities, symptom burden, medication adherence attitudes, emotional well-being, cognitive functioning, pain experiences, and social support.

Key instruments administered included the PROMIS Global Health Scale, Self-Administered Comorbidity Measure (SACM), Breast Cancer Prevention Trial Symptom Checklist (BCPTC), Fatigue Symptom Inventory (FSI), and Patient Satisfaction with Cancer Care (PSCC), among others.

Demographic and clinical information—including age, sex, gender identity, race/ethnicity, education, employment status, cancer diagnosis details, and endocrine therapy use—was also collected. A full list of questionnaires and quality control details is provided in Appendix C.

#### 3.3.4 MEMS Data.

As part of the study, the participants were asked to use a pill bottle known as the RxCap and place their breast cancer endocrine therapy pills into the RxCap. RxCap is a smart, Bluetooth-connected pill bottle that records the timing of bottle-opening, which is considered a proxy for medication-taking in our study. The RxCap device also had a smartphone application called the RxCap Engage. The participants were recommended to sync the device with the app once a week. Thus, the RxCap recorded the intake of once daily prescribed endocrine therapy and was used to determine the ground-truth label for daily adherence status.

## 4 MULTIMODAL MODELING OF MEDICATION ADHERENCE

In this study, we considered four distinct input modalities: Fitbit sensor data, ecological momentary assessment (EMA) data collected via the Sensus smartphone application [51], medication adherence logs derived from MEMS caps, and baseline survey responses collected at enrollment. Each modality contributed complementary behavioral, physiological, or psychological signals relevant to adherence prediction.

Among these modalities, our primary interpretability analyses focused on Fitbit and EMA data, due to their high temporal resolution, real-time availability, and low participant burden-qualities that make them particularly well-suited for supporting personalized interventions. In contrast, baseline survey data provided valuable contextual insights (e.g., medication beliefs, side effect profiles), but their one-time collection at study enrollment limited their utility for modeling dynamic, day-level adherence patterns. To capture their predictive value, we employed tree-based regression models (Random Forest, XGBoost, LightGBM) on baseline features to generate static adherence probabilities.

In the context of MEMS, although it served as the primary source of objective adherence ground truth, detailed modeling of MEMS-derived data has been previously explored by Kaur et al. [27] using LSTM-based frameworks. Building on this foundation, we extended the literature by incorporating a novel SHAP-based interpretability analysis of our MEMS LSTM models (Figure 4). This analysis revealed temporal patterns in dosing behavior and time-of-day effects, offering new insights into the behavioral regularities that underpin adherence.

For sequential modeling, we selected the LSTM network as our primary architecture due to its capacity to capture short- to medium-term dependencies in temporally irregular and noisy health data. To support this design choice, we conducted comparative evaluations with two additional deep sequence models-Transformer and Temporal Convolutional Networks (TCNs)-using identical time-series inputs across all modalities. Results from these comparisons are provided in Appendix E. While Transformer and TCN models demonstrated competitive performance in selected configurations, LSTM consistently matched or outperformed them, particularly in the EMA and MEMS modalities. These findings, along with LSTM’s robustness in small to medium-sized datasets, supported its use as the primary model in our main analysis.

In addition, to establish baseline comparisons with simpler models, we trained logistic regression (LR) and random forest (RF) classifiers using aggregated features derived from the time-series data. As summarized in Appendix D, these models exhibited substantially lower performance compared to the deep sequence models, underscoring the importance of capturing temporal dynamics in adherence prediction.

**Fig. 1.**
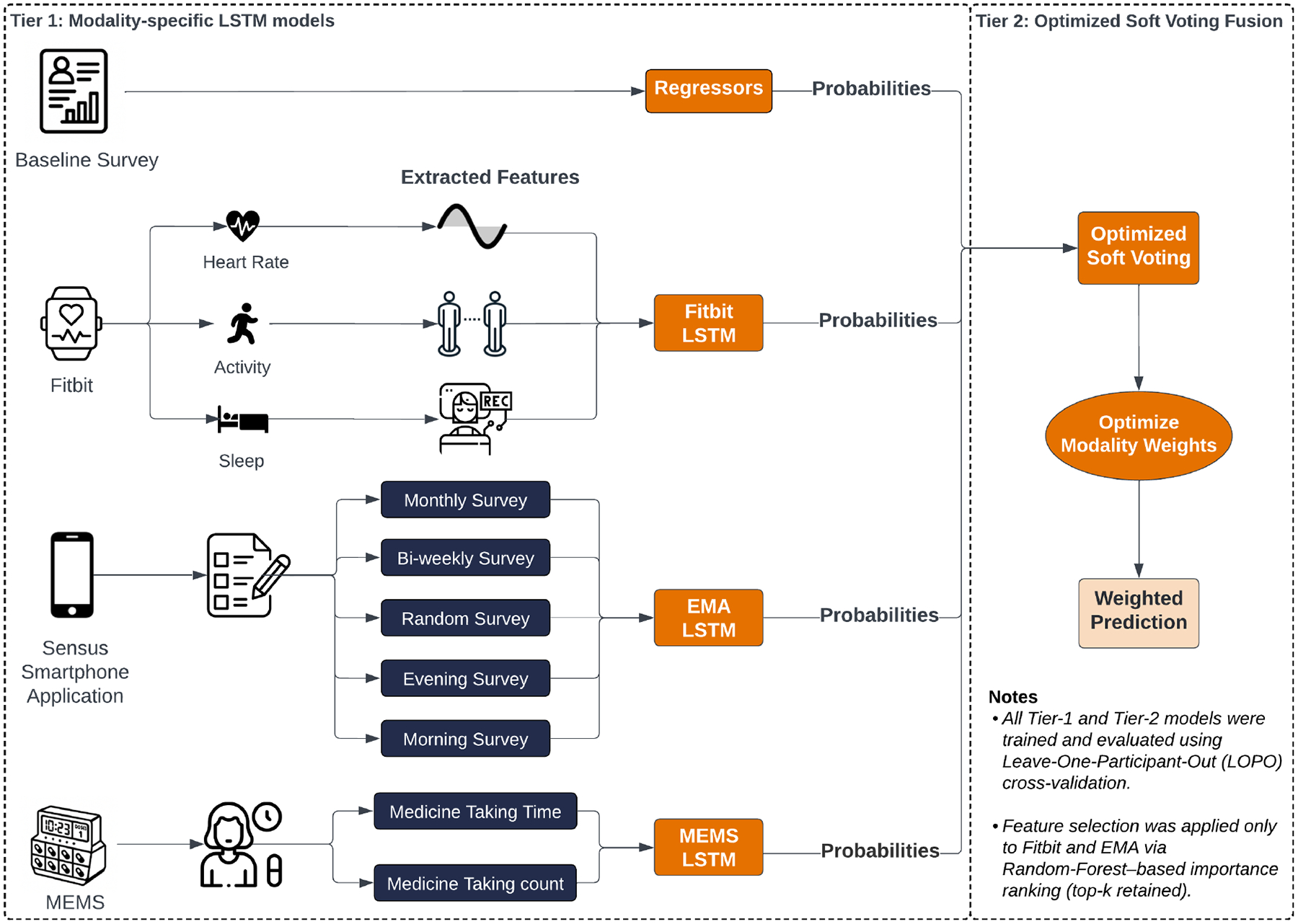
Schematic of the two-tier modeling framework: modality-specific models (Tier 1) feed into an optimized soft-voting fusion mechanism (Tier 2).

Figure 1 illustrates our two-tier modeling framework. In Tier 1, we trained separate modality-specific models for Fitbit, EMA, MEMS, and survey data. In Tier 2, we employed optimized soft voting to fuse the outputs of these individual models and generate a final adherence probability.

### 4.1 Feature Engineering

Each data modality was first preprocessed, including imputation to address missingness. Subsequently, daily-level features were derived for the adherence prediction task:

- **EMA:** Daily scores were extracted from self-reported responses that captured not only psychological states—such as stress and emotion—but also social interactions, perceived environmental conditions, and contextual factors relevant to daily well-being.
- **Fitbit:** Features included total steps, minutes of physical activity, sleep duration, heart rate variability metrics, and circadian rhythm [52] indicators computed from steps and heart rate patterns.
- **MEMS:** Daily binary adherence labels (taken vs. missed) were recorded, along with contextual information such as dosing time and adherence timing regularity.
- **Baseline Survey:** Less frequently changing psychosocial and behavioral measures were collected at baseline, including assessments of health beliefs, medication attitudes, self-efficacy, and perceived barriers to adherence.

All daily features—excluding those from the Baseline Survey modality—were concatenated into temporal sequences spanning 2 to 7 days, serving as input to modality-specific LSTM networks (Tier 1) tasked with predicting next-day adherence. This sequential representation enabled the models to capture short-term temporal dependencies and behavioral dynamics.

### 4.2 Feature Selection

Feature selection and hyperparameter tuning were conducted within each inner fold of the nested cross-validation framework to ensure an unbiased evaluation.

For each inner training split, a Random Forest classifier was trained using the most recent day’s features to compute feature importances and rank predictors. Candidate feature subsets were defined based on these rankings. A grid search was then performed to jointly optimize (1) the number of selected features and (2) LSTM hyperparameters (e.g., number of units, dropout rate, *ℓ*_2_ regularization coefficient). Selected features were consistently applied across all time steps in the input sequence.

Performance during tuning was evaluated using balanced accuracy on inner validation splits. The best feature subset and hyperparameters were then used to train the final model on the complete inner training set and evaluated on the corresponding outer fold, maintaining strict participant-level data separation.

### 4.3 Tier 1: Modality-specific Models

#### 4.3.1 Model Development and Evaluation Setup.

In Tier 1, separate LSTM models were trained for each modality (Fitbit, EMA, and MEMS) using sequential inputs spanning 2 to 7 days. A LOPO cross-validation strategy was employed: in each fold, one participant’s data was held out for testing, while the remainder formed the training set.

For the Fitbit and EMA modalities, which contained higher-dimensional and heterogeneous features, we applied the feature selection and model tuning procedure described in Section 4.2.

All steps—feature selection, hyperparameter tuning, and model training—were performed exclusively on the training participants within each fold to prevent information leakage and ensure participant-independent generalization.

The optimal feature subset and hyperparameter configuration, determined from the training participants, were then used to train a final model, which was evaluated on the held-out test participant.

For the MEMS modality, which comprises low-dimensional binary adherence indicators and time-of-day dosing features, no additional feature selection was performed due to its compact and interpretable nature.

#### 4.3.2 Model Performance and Feature Interpretation.

The predictive performance of modality-specific LSTM models across different time window sizes (2 to 7 days), along with 95% confidence intervals (CIs), is summarized in Table 2.

Among all modalities, MEMS consistently achieved the highest Macro BA and Macro F1, with relatively narrow CIs, indicating both superior and stable performance. EMA models performed second best, followed by Fitbit.

Notably, extending the temporal input beyond three days did not lead to significant gains and in some cases resulted in marginal performance degradation. This suggests that next-day adherence is primarily influenced by recent behavioral patterns, and longer historical windows may introduce noise or reduce generalizability.

We employed macro-averaged Balanced Accuracy and macro-averaged F1 as evaluation metrics to account for potential class imbalance:

(1) $$\text{Macro-Averaged}\;\text{Balanced}\;\text{Accuracy}=\frac{1}{C}\sum_{i=1}^{C}\frac{1}{2}\left(\frac{{\text{TP}}_{i}}{{\text{TP}}_{i}+{\text{FN}}_{i}}+\frac{{\text{TN}}_{i}}{{\text{TN}}_{i}+{\text{FP}}_{i}}\right)$$

(2) $$\text{Macro-Averaged}\;\text{F}1=\frac{1}{C}\sum_{i=1}^{C}F1_{i}$$

In Tier 1, MEMS-based models consistently achieved the highest predictive performance (BA=0.73, F1=0.84), aligning with prior studies highlighting the effectiveness of direct electronic adherence monitoring [10]. EMA-based models demonstrated moderate performance (BA=0.64) [14, 42], and Fitbit-based models achieved the lowest predictive capability (BA=0.62) [39].

Extending the input time window beyond three days did not consistently enhance performance across modalities, reinforcing that proximal behavioral and psychological states are key predictors of immediate adherence.

**Fig. 2.**
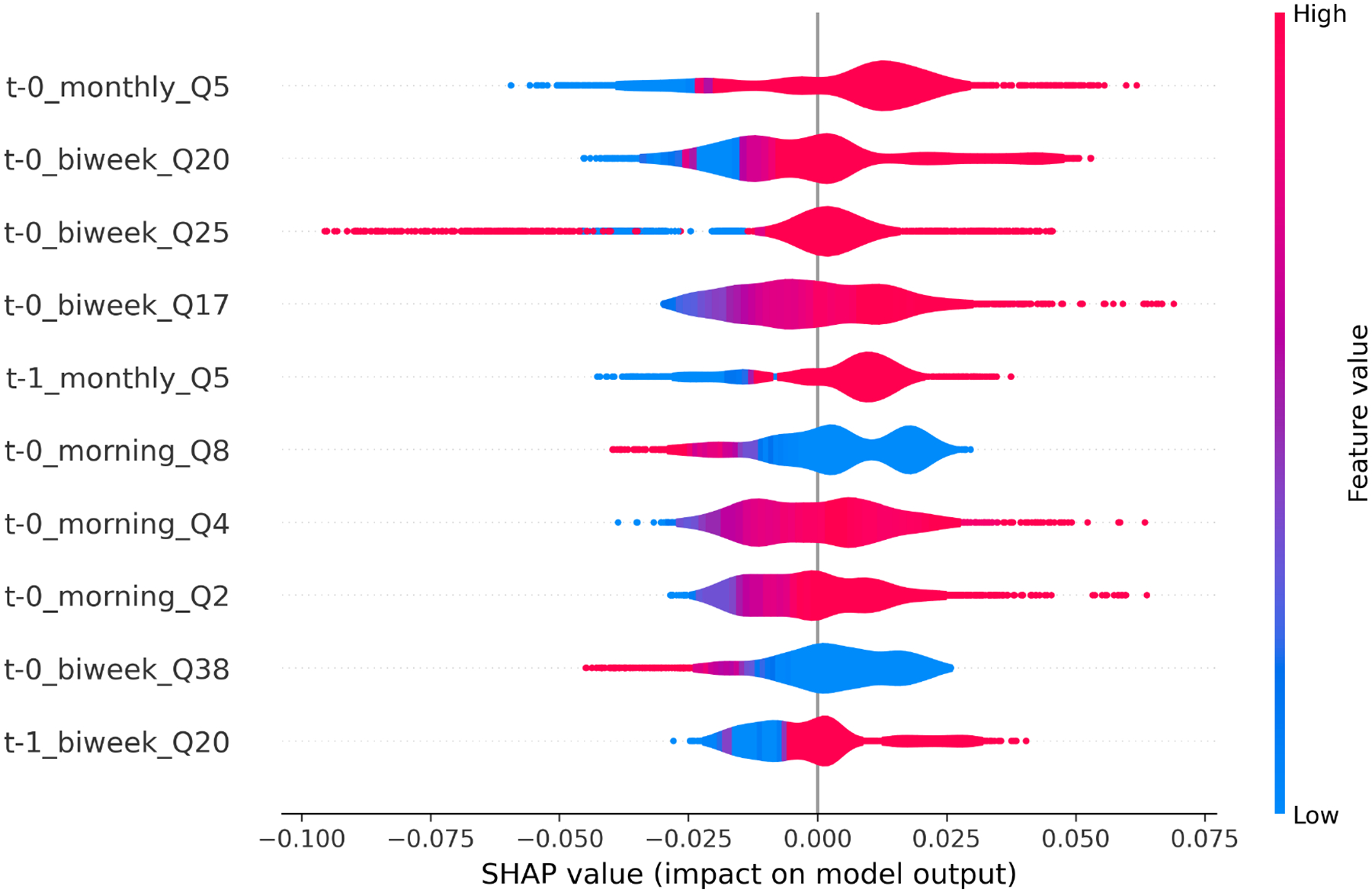
Top 10 EMA Features Contributing to Adherence Prediction.

*EMA LSTM Model Interpretation*. To interpret the decision mechanisms underlying the EMA-based LSTM model trained with a time window of 3, we conducted SHAP analysis using a LOPO cross-validation framework. For each fold, we computed SHAP values based on the top 50 EMA features selected from the training set using inner-fold feature selection. The final SHAP violin plot (Figure 2) aggregates the top 10 most impactful features across all folds. Here are the feature descriptions:

- **monthly_Q5:** “Do you have someone to run errands if you need it?”
- **biweek_Q20:** “Joint pains?”
- **biweek_Q25:** “Forgot to take medicine in past 2 weeks?”
- **biweek_Q17:** “Bothered by vaginal dryness?”
- **monthly_Q5:** “Do you have someone to run errands if you need it?” (previous day)
- **morning_Q8:** “How sad are you right now?”
- **morning_Q4:** “How excited are you right now?”
- **morning_Q2:** “How anxious are you right now?”
- **biweek_Q38:** “Not able to stop/control worrying?”

*Summary of EMA Feature Importance Patterns*. The SHAP analysis identified both psychological and physical health variables, assessed via EMA surveys, as key contributors to adherence prediction. Several consistent themes emerged:

- **Self-Reported Adherence:** The most impactful feature, “Forgot to take medicine in past 2 weeks?”, exhibited a strong positive SHAP association—higher values (indicating forgetfulness) corresponded to lower predicted adherence.
- **Side Effect Burden:** Joint pain and vaginal dryness showed moderate importance, with high symptom reports generally reducing adherence probabilities.
- **Mood:** Morning emotional states such as sadness, anxiety, and excitement contributed directionally. Higher sadness or anxiety often decreased adherence predictions, whereas excitement had more mixed associations.
- **Social Support:** The presence of social support generally promoted adherence, indicating that instrumental support may facilitate consistent medication-taking behaviors.
- **Control of Worry:** Being able to control or stop worry displayed directionally mixed effects—greater worry control appeared to inhibit medication adherence for some and promote adherence for others.

These findings highlight the importance of both behavioral and psychosocial EMA signals in daily adherence modeling. Morning emotions, perceived side effects, and access to social support jointly shape medication-taking behavior. Future adaptive interventions may benefit from tailoring reminders or supports based on an individual’s side effect burden and moods.

**Fig. 3.**
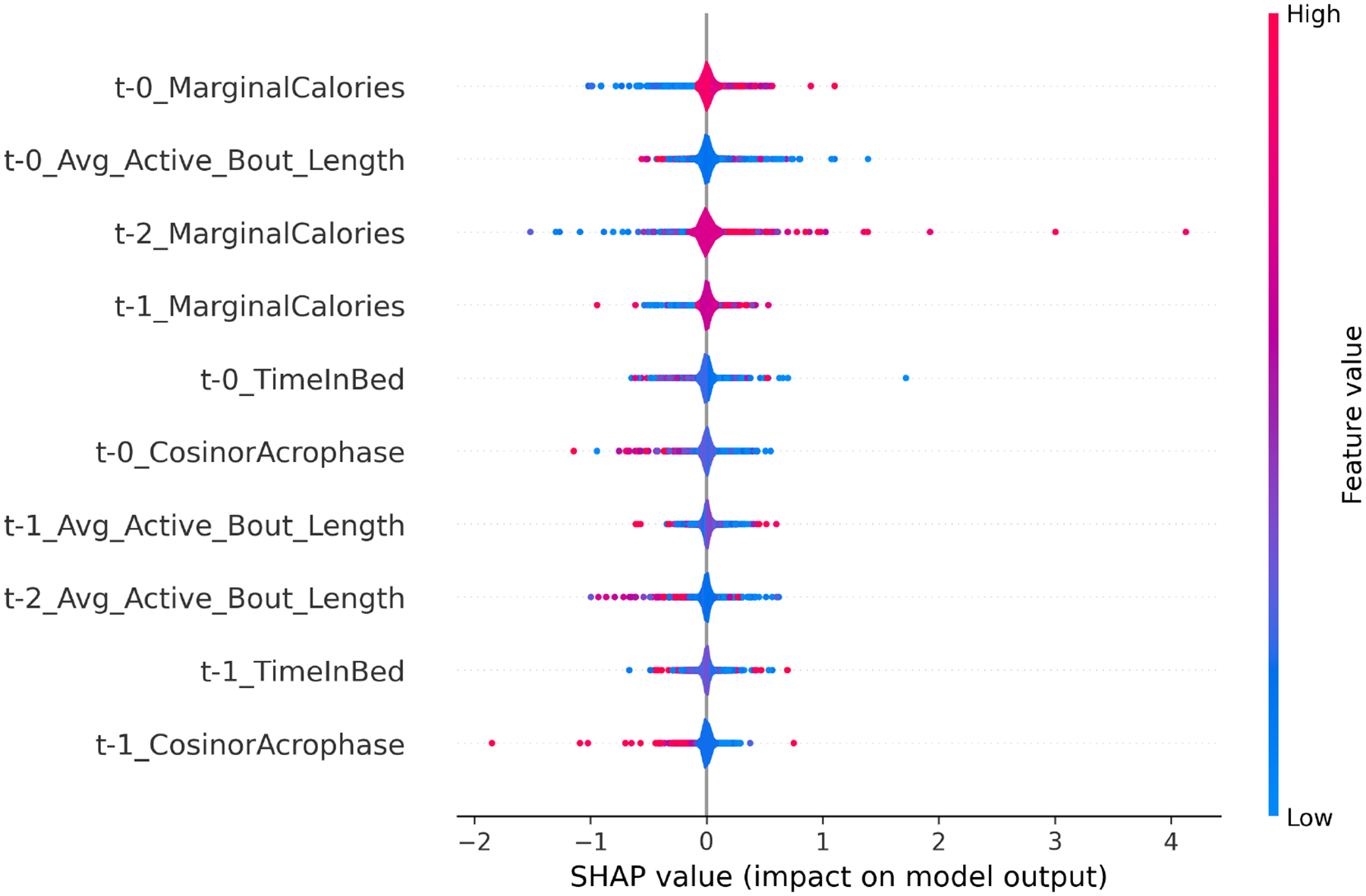
Top Fitbit Features Contributing to Adherence Prediction

*Fitbit LSTM Model Interpretation*. Similarly, SHAP analysis on Fitbit-based models indicated that physical activity structure and physiological regulation metrics were key predictors. The final summary plot in Figure 3 displays the 10 most impactful features across all folds. Appendix B provides a description of each feature.

*Summary of Fitbit Feature Importance Patterns*. The SHAP summary plot revealed that both physical exertion metrics (e.g., MarginalCalories, Avg Active Bout Length) and circadian rhythm indicators (e.g., CosinorAcrophase, TimeInBed) were highly predictive of daily medication adherence:

- **Energy Expenditure:** *MarginalCalories* across all three days emerged as strong positive contributors—higher values generally increased predicted adherence. This suggests that greater energy output may reflect higher daytime engagement or structured routines that support adherence.
- **Sustained Physical Activity:** *Avg Active Bout Length* also appeared consistently across all three days. However, its effects were more heterogeneous, implying that while some individuals benefit from sustained activity patterns (e.g., through behavioral consistency), for others, physical fatigue may reduce likelihood of medication-taking.
- **Sleep and Circadian Regulation:** *TimeInBed* and *CosinorAcrophase* showed moderate but consistent influence on model predictions. Longer time in bed generally promoted adherence, possibly reflecting better rest and recovery. Meanwhile, *CosinorAcrophase*—which refers to the timing of the peak in daily step rhythms as estimated through cosinor analysis—exhibited both positive and negative SHAP values. This suggests that delayed or misaligned circadian peaks may interfere with self-care routines in some participants.

Overall, the Fitbit-based model captures both behavioral regularity and circadian structure as important correlates of adherence. These findings underscore the dual importance of maintaining structured activity rhythms and ensuring adequate recovery (via sleep) in supporting adherence behaviors. Yet, individual variability in SHAP directionality further emphasizes the need for personalized modeling approaches when applying wearable data to behavioral prediction tasks.

**Fig. 4.**
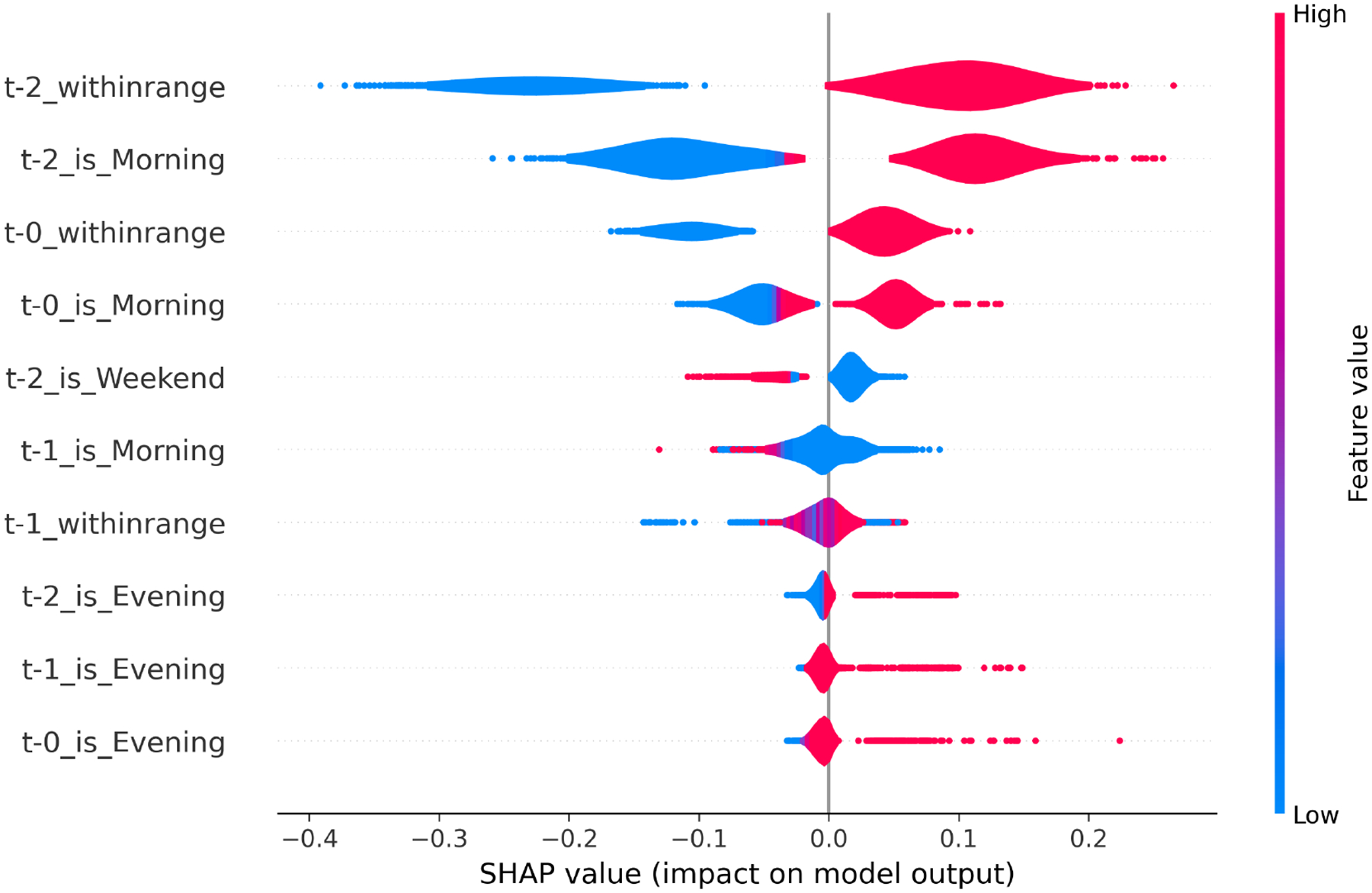
Top MEMS Features Contributing to Adherence Prediction

*MEMS LSTM Model Interpretation*. SHAP analysis on the MEMS-based LSTM model further revealed the key temporal behavioral signals associated with medication adherence. Figure 4 presents the top 10 contributing features based on SHAP values aggregated across all folds.

*Summary of MEMS Feature Importance Patterns*. The SHAP summary plot of the MEMS-based LSTM model revealed that recent adherence history and contextual timing cues were the most influential predictors of next-day medication behavior. Several interpretable patterns emerged:

- **Recent Adherence Behavior:** The model assigned strong positive importance to consistent adherence in the previous two to three days. This finding reinforces the notion that medication-taking behavior exhibits short-term continuity—patients who took their medication recently were more likely to continue doing so.
- **Morning Dosing Patterns:** Morning intake events were among the most predictive temporal features. Adherence behavior tied to morning routines tended to increase the model’s confidence in future adherence, suggesting that structured morning habits may support medication regularity.
- **Weekend Disruptions:** Adherence behavior occurring on or after weekends was associated with lower predicted adherence probabilities. This likely reflects the destabilizing effect of weekend routines, where typical daily structures may be interrupted.
- **Evening Dose Variability:** Features related to evening medication-taking showed mixed contributions. In some instances, evening doses were predictive of continued adherence; in others, they appeared to correspond with decreased likelihood—possibly due to fatigue, end-of-day distractions, or reduced consistency in evening routines.

These findings highlight the importance of recent behavioral patterns and temporal context in shaping adherence predictions. The model effectively leveraged short-term memory of medication behavior and time-of-day regularity, offering a strong foundation for time-sensitive and habit-aware intervention strategies.

### 4.4 Tier 2: Optimized Soft Voting Fusion

#### 4.4.1 Model Development and Evaluation Setup.

In Tier 2 of our framework, we integrated the predicted adherence probabilities generated by the modality-specific LSTM models from Tier 1 using a soft-voting fusion strategy. For each participant-day instance, predictions from different modalities (Fitbit, EMA, and MEMS) were aligned by participant ID and date to ensure temporal and identity consistency across streams.

Rather than training a separate meta-learner, we employed a grid search-based soft-voting mechanism to optimize fusion weights in a participant-independent manner. Specifically, within each LOPO fold, we optimized a set of modality-specific fusion weights {*w*_1_*, w*_2_*, …, w*_*M*_} based solely on the training participants. The fused probability was computed as:

(3) $$\text{FusedProb}=\sum_{m=1}^{M}w_{m}\cdot {\text{Prob}}_{m},\;\text{subject}\;\text{to}\;\sum_{m=1}^{M}w_{m}=1.$$

where Prob_*m*_ denotes the predicted adherence probability from modality *m*, and *w*_*m*_ is its associated weight. Fusion parameters were selected to maximize the macro-averaged balanced accuracy on the training participants, ensuring that test predictions remained strictly participant-independent.

We evaluated three fusion baselines:

- **Majority Baseline:** A non-informative reference model that always predicts the most frequent class. Since one class is entirely ignored, this results in a theoretical macro-averaged balanced accuracy and macro F1-score of 0.50, regardless of class distribution.
- **Baseline Soft Voting:** Uniformly weighted fusion of modality-specific predictions (*w*_*m*_ = 1/*M* for all *m*), without optimization.
- **Optimized Soft Voting:** Grid search-optimized fusion weights tailored within each LOPO fold.

#### 4.4.2 Model Performance and Interpretation.

Fusion performance across different modality combinations and input window sizes (2–7 days), along with 95% CIs, is summarized in Table 3. For each configuration, macro-averaged BA and macro-averaged F1 with 95% CIs are reported, and the best-performing strategies are highlighted in bold.

The optimized soft-voting fusion in Tier 2 showed significant benefits for combinations involving MEMS data. MEMS combined with EMA or Fitbit substantially improved adherence prediction compared to uniform-weight baselines, with both higher mean performance and narrower CIs, indicating that direct adherence measurements significantly enhance the value of secondary behavioral and physiological data. Additionally, the fusion of EMA and Fitbit data alone also demonstrated promising and stable predictive performance, with 95% CIs consistently above baseline levels, highlighting the potential value of purely mobile-sensed behavioral and physiological modalities. This result aligns with prior literature emphasizing the viability of mobile sensing data streams, such as self-reported symptoms and wearable-derived metrics, for reliably capturing health-related behaviors, including medication adherence [7, 39, 42].

To further explore the contribution of less-frequently changing individual characteristics, we incorporated an additional modality—baseline survey and demographic features—into the soft-voting fusion framework. As reported in Table 3, this addition led to only marginal improvements in predictive performance across most time windows. The best result was observed under the 2-day input window (BA=0.84 [95% CI: 0.80–0.88], F1=0.88 [95% CI: 0.85–0.92]), but performance gains quickly diminished as the input horizon extended. These findings suggest that while baseline information may provide a slight enhancement when combined with time-series modalities, it does not meaningfully influence model predictions in most cases. Furthermore, the overlapping CIs between fusion models with and without baseline information indicate that this static data source does not contribute significantly beyond behavioral and physiological signals. This reinforces the interpretation that short-term adherence behavior is predominantly driven by proximal behavioral, social, psychological, and physiological signals, rather than by static or background characteristics.

Our findings thus reinforced the utility of integrating mobile sensing data into adherence monitoring systems, particularly in clinical scenarios where MEMS data may not be feasible or consistently available. Even without MEMS, multimodal fusions involving EMA and Fitbit yielded competitive performance with tight CIs, underscoring the potential of passive, scalable sensing approaches for adherence prediction.

*Perturbation-based Interpretation*. The comparative performance patterns observed in Tier 2 naturally provide a perturbation-based interpretation of modality importance. To quantify these effects, we systematically compared the best-performing Tier 1 LSTM models for each single modality—Fitbit, EMA, and MEMS—against the optimized fusion results. As shown in Table 4, all performance values are reported alongside 95% CIs to reflect both central tendencies and the stability of each configuration.

Several important insights emerged from these comparisons:

First, removing MEMS (i.e., relying solely on EMA+Fitbit fusion) resulted in a moderate decline in performance relative to MEMS-inclusive combinations. Specifically, EMA+Fitbit achieved a balanced accuracy (BA) of 0.65 and an F1-score of 0.68, compared to 0.73 (BA) and 0.84 (F1) for MEMS+EMA, and 0.79 (BA) and 0.88 (F1) for MEMS+Fitbit. These results confirm that prior MEMS data offers a uniquely strong predictive signal. Nonetheless, the EMA+Fitbit fusion substantially outperformed the majority baseline (BA = 0.50), and closely matched the performance of EMA alone (BA = 0.64, F1 = 0.66). Collectively, these findings highlight the value of mobile sensing modalities—Fitbit-derived physiological patterns and EMA-reported psychological states—particularly in scenarios where MEMS deployment may be impractical or unavailable.

Second, although Fitbit alone underperformed relative to other modalities (BA = 0.62, F1 = 0.66), combining it with MEMS (BA = 0.79, F1 = 0.88) or with EMA+MEMS (BA = 0.83, F1 = 0.88) yielded substantial gains. This suggests that Fitbit contributed complementary physiological signals not captured by EMA or MEMS alone.

Third, incorporating baseline survey and demographic features into the full multimodal fusion resulted in only marginal performance improvements. Balanced accuracy increased slightly from 0.83 (EMA+Fitbit+MEMS) to 0.84 with the addition of survey data, while the F1-score remained stable at 0.88. Notably, the 95% confidence interval for F1 narrowed modestly from [0.84, 0.91] to [0.85, 0.92]. These results reinforce the conclusion that long-term, trait-level characteristics offer limited incremental value for short-term adherence prediction relative to dynamic behavioral and physiological signals. We hypothesize that much of the predictive signal from baseline traits is already indirectly captured through EMA-reported psychological states and Fitbit-derived physiological patterns, reducing the added benefit of explicitly including survey data.

Notably, the combination of Fitbit and MEMS—both passively collected data sources—achieved high predictive accuracy (BA=0.79, F1=0.88), approaching that of full multimodal fusion. This demonstrated that effective adherence prediction was feasible even in the absence of active self-reported input (i.e., EMA). Such systems were particularly promising for real-world deployment scenarios, where reducing participant burden and ensuring passive, unobtrusive monitoring were critical to sustained engagement and scalability.

Finally, the highest performance was consistently achieved by fusing all three core modalities—EMA, Fitbit, and MEMS—underscoring the synergistic benefits of integrating behavioral, physiological, and direct adherence-monitoring signals.

Overall, our Tier 2 experiments functioned as a natural perturbation-based ablation analysis, revealing the unique and complementary value of each modality without requiring explicit removal experiments. These results highlight the promise of multimodal mobile sensing frameworks for enabling scalable, low-burden monitoring of medication adherence in real-world settings.

## 5 DISCUSSION

### 5.1 Summary of Findings

This study presented a comprehensive evaluation of medication adherence prediction using multimodal time-series data, integrating Fitbit-derived physiological signals, EMA-reported psychological, social, and environmental states, and MEMS-recorded adherence behavior. Several key findings emerged from our two-tier predictive modeling framework:

1. *MEMS-based Models Achieved the Highest Predictive Accuracy*. Across all time windows, MEMS-based LSTM models consistently achieved the highest macro-averaged balanced accuracy (BA = 0.73) and F1-score (F1 = 0.84), reinforcing the value of past adherence behavior being the most influential on next-day adherence.
2. *Self-reports from EMA Captured Social, Affective, and Behavioral Correlates of Adherence*. EMA-based models outperformed Fitbit-only models (peak BA = 0.64), suggesting that self-reported psychological and behavioral factors offered meaningful insight into daily adherence behaviors [42]. SHAP analysis indicated that variables such as sadness, anxiety, forgetfulness, and perceived social support were among the most influential features.
3. *Fitbit-derived Features Provided Complementary Behavioral Signals*. Although Fitbit-only models yielded lower predictive accuracy, the integration of Fitbit data with EMA and/or MEMS led to significant gains. Physiological indicators—such as physical activity, sleep patterns, and circadian rhythm regularity—captured behavioral aspects not fully represented in other modalities [7, 39].
4. *Multimodal Fusion Outperformed Single-modality Models*. Tier 2 fusion models that combined MEMS, EMA, Fitbit, and baseline survey data achieved the highest overall performance (BA = 0.84, F1 = 0.88). Notably, EMA+Fitbit fusion (excluding MEMS) still outperformed both baseline and individual modality models, demonstrating the potential of non-invasive mobile sensing for adherence prediction when MEMS is unavailable.
5. *Passive Sensing Alone Enabled Strong Predictive Performance*. The combination of Fitbit and MEMS—both passively collected sources—produced high predictive accuracy (BA = 0.79, F1 = 0.88), comparable to full multimodal fusion. These results highlight the feasibility of passive-sensing-based systems for real-world deployment, particularly where minimizing participant burden and maximizing scalability are critical.
6. *Proximal Behavioral Signals Were Most Predictive*. Models trained on shorter temporal windows (2–3 days) consistently outperformed those using longer input sequences, indicating that recent behavioral and psychological states exerted the strongest influence on next-day adherence. This finding aligns with theories of short-term behavioral regulation [45].
7. *Contributions to the Field*. This study made several novel contributions to the field of adherence prediction and mobile health monitoring. First, it provided empirical evidence that combining EMA with passively collected physiological data (Fitbit) could reliably predict daily medication adherence, even in the absence of MEMS-based electronic monitoring. This expanded the possibilities for low-burden, scalable adherence monitoring in real-world and underserved settings.
  Second, the study introduced a flexible two-tier modeling framework that supported both modality-specific representation learning and cross-modal fusion, demonstrating how complementary signals from behavioral, physiological, and adherence data could be integrated for enhanced prediction.
  Third, the results highlighted the temporal specificity of adherence-relevant signals—showing that recent psychological and physiological states, rather than static baseline traits, were the strongest predictors of near-term adherence behavior. This insight informed the design of personalized, dynamic intervention systems that were responsive to short-term fluctuations in patient state.
  Collectively, these contributions advanced the methodological toolkit for digital adherence monitoring and offered actionable insights for the development of next-generation just-in-time adaptive interventions (JITAIs) in oncology and beyond.
8. *Implications for Future Intervention Design*. These findings have direct implications for the development of low-burden, scalable adherence interventions. Notably, meaningful predictive accuracy was achieved even in the absence of MEMS data by fusing EMA and Fitbit inputs—supporting the feasibility of personalized, digital interventions to promote adherence [35].

In sum, our results underscore the value of integrating behavioral, physiological, and electronic adherence signals within a unified modeling framework. The proposed approach not only advances methodological rigor in adherence prediction but also provides actionable insights for designing scalable, interpretable digital health systems capable of supporting real-time interventions.

### 5.2 Clinical Implications

Our findings suggest several important implications for clinical practice and digital health system design. The strong performance of MEMS-based models reinforces the value of direct electronic adherence monitoring for high-fidelity tracking and timely identification of patients at risk for non-adherence—particularly those managing complex regimens or with historically low adherence [10, 49]. Enhancing MEMS data with behavioral and physiological signals from EMA and Fitbit added explanatory power, offering insight into why non-adherence occurs—such as emotional distress, fatigue, or disrupted routines—thereby supporting more personalized and context-aware interventions. At the same time, the minimal added value of static baseline data suggests that recent, dynamic signals are more relevant for predicting day-level adherence. Together, these results underscore the importance of continuous sensing and real-time monitoring to enable individualized, adaptive support in everyday care.

*Informing Personalized Interventions*. To move from prediction to action, one implementation pathway involves using the real-time output of our multimodal adherence model as a trigger within a Just-In-Time Adaptive Intervention (JITAI) framework. For instance, if the model detects elevated non-adherence risk based on a combination of high EMA-reported distress and reduced Fitbit-recorded sleep or activity, the system could deliver an immediate, context-sensitive intervention—such as a supportive adherence prompt, a motivational message, or a coping strategy tailored to the user’s symptom profile (e.g., fatigue or anxiety). These interventions could be delivered through a smartphone app, smartwatch notification, or text message and calibrated by historical response data. For lower-burden deployments without EMA, physiological signals (e.g., heart rate variability, sleep disruption) could trigger automated check-ins or suggest routine-stabilizing behaviors. Future work could integrate reinforcement learning that maps risk patterns to intervention type, timing, and intensity, supporting scalable and personalized adherence support.

Notably, these combinations illustrate that meaningful adherence prediction is feasible even without MEMS data, opening up opportunities for lower-cost, scalable solutions in underserved or resource-limited settings. Because both Fitbit and MEMS data streams are passively collected and require no direct user input, they are especially well-suited for long-term, low-burden deployment in real-world contexts.

Finally, incorporating multimodal adherence predictions into clinical workflows could enhance patient-clinician communication and enable more strategic, macro-level interventions targeting adherence. By surfacing interpretable risk signals based on daily behavioral and physiological patterns, these longitudinal models may facilitate shared decision-making, strengthen patient engagement, and improve the therapeutic alliance between providers and patients.

### 5.3 Considerations for Equity and Future Deployment

Translating predictive adherence models into real-world clinical and behavioral settings necessitates careful consideration of feasibility, equity, and ethical safeguards. While this study was conducted under controlled research conditions, its modeling and sensing infrastructure was developed with practical deployment constraints in mind.

*Equity and Fairness in Real-world Use*. Equitable deployment of adherence prediction systems requires careful attention to disparities in technology access, digital literacy, and algorithmic performance. In addition to LOPO validation and individualized interpretability (e.g., SHAP)—to support transparency and personalization, we conducted a preliminary subgroup sensitivity check by age (<50 years vs. ≥50 years; 3-day input window). We observed no systematic degradation in macro-averaged balanced accuracy across age groups; full subgroup results with 95% CIs and group-wise data summaries are provided in Appendix F. Future work should expand these efforts to assess performance across additional demographic variables (e.g., race, education), apply fairness metrics such as group-wise calibration, and incorporate participatory co-design practices to ensure equitable utility of model outputs and interventions across diverse populations.

*Toward Online, Real-Time Deployment*. Transitioning from retrospective modeling to real-time intervention delivery introduces several practical challenges for ubiquitous systems. These include model drift over time, inference latency, robustness to missing or delayed data, and the need for efficient architectures capable of on-device or near-device execution. To support personalized interventions in everyday contexts, future deployments will need to address these constraints while maintaining interpretability, privacy, and low participant burden—key factors for integration into clinical workflows and long-term adherence support.

*Privacy and Participant Acceptability*. Strong privacy protections and sustained participant trust are prerequisites for large-scale multimodal adherence monitoring. In this study, all data collection was approved by the IRB, obtained with explicit informed consent, and securely transmitted and stored on encrypted, access-controlled university servers. Future clinical implementations will require comparable safeguards, including end-to-end encryption, role-based access control, and routine de-identification of personally identifiable information. Equally important, participants must perceive long-term passive monitoring as acceptable and minimally intrusive. During our six-month pilot, participants generally reported comfort with wearable and EMA data collection; however, expectations and privacy concerns may differ in larger and more diverse cohorts. Future deployments should therefore integrate ongoing user feedback, transparent communication of data use, and accessible mechanisms for participants to pause or review their data. Such measures are essential to ensure that the benefits of timely, just-in-time adherence interventions can be realized without compromising privacy or trust.

### 5.4 Limitations and Future Work

This study introduced a multimodal adherence prediction framework with several methodological strengths, including LOPO cross-validation for participant-independent generalization, robust feature selection and fusion optimization, and tiered model evaluation. Still, several limitations warrant discussion. The modest sample size (N = 20) and demographic homogeneity (80% White, all U.S.-based) limit generalizability. Future work should evaluate these models in larger and more diverse cohorts to assess cross-context robustness and subgroup variability in adherence predictors.

Our current modeling approach captures short-term behavioral variation using recent multimodal inputs (e.g., past 2–7 days), but it assumes relative stationarity within those windows. Yet adherence patterns often shift over longer periods due to treatment changes, symptom evolution, or psychosocial factors. Future modeling efforts should explore more adaptive temporal frameworks—such as time-aware LSTMs, sliding-window recalibration, or online learning—to better track longitudinal behavioral transitions and dynamically identify high-risk periods.

While the fusion strategy provided interpretability at the modality level, finer-grained explanation remains an open area. Integrating feature-level interpretation techniques (e.g., SHAP, permutation importance) could enhance transparency and offer more actionable insights about the psychological, social, or physiological states driving non-adherence. Such insights may inform the design of precision-tailored interventions.

This work focused on next-day adherence prediction, supporting just-in-time intervention use cases. However, long-term adherence maintenance remains critical in chronic care. Extending prediction horizons to weekly or monthly windows may support more strategic planning, including escalation protocols or longitudinal treatment adjustments.

Concerns around fairness and equity are essential as these models move toward clinical deployment. In this study, a preliminary age-based subgroup sensitivity analysis (Appendix F) showed comparable macro-averaged balanced accuracy across age groups. Higher F1 in the 50 group appeared driven by differences in class balance and the common decision threshold, rather than true age-related model bias. Future work with larger, more diverse cohorts should examine model performance across race, education, and digital literacy; adjust calibration and thresholds for each subgroup; and test bias-mitigation methods to ensure fair and reliable results.

Relatedly, although our dataset already reflected natural missingness due to non-wear and skipped surveys, we conducted additional stress-test analyses in which EMA and Fitbit features were artificially removed under both missing completely at random (10–50% MCAR at the feature level) and structured patterns (simulated 3-day gaps in 10% of weeks). Predictive performance remained remarkably stable across conditions: balanced accuracy and AUC declined by < 0.02 on average relative to baseline, and Brier scores were unaffected. These results indicate that the proposed framework is robust to missing data. Full details and sensitivity results are provided in Appendix G.

Finally, while Fitbit and EMA models performed competitively, MEMS-based models consistently outperformed them. Bridging this performance gap through improved passive sensing modalities, richer multimodal representations, and more expressive modeling architectures remains a promising direction. Additional data streams—such as GPS, ambient audio, or social interaction metrics—could further strengthen prediction accuracy and facilitate low-burden, scalable adherence monitoring for real-world use.

## 6 CONCLUSION

This study introduced a two-tier framework for predicting daily medication adherence using multimodal mobile sensing data from Fitbit, EMA, and MEMS. Modality-specific LSTM models captured temporal dynamics, and a soft-voting fusion approach improved prediction over unimodal baselines. MEMS provided the strongest standalone signal, while EMA and Fitbit offered valuable psychological and physiological context. Even without MEMS, EMA+Fitbit models achieved competitive accuracy, highlighting the feasibility of low-burden, passive monitoring.

Baseline demographic data added minimal predictive value, reinforcing the importance of recent, dynamic signals. These findings support the use of mobile sensing to inform personalized, just-in-time interventions and enable scalable adherence support in clinical and resource-limited settings.

Future work should explore longer prediction horizons, adaptive temporal modeling, and individualized explainability to improve clinical relevance and model trustworthiness.

## Figures and Tables

**Table 1.** Demographic and Clinical Characteristics of Participants.

Characteristic	Number (Percent)
**Age in Years**	Mean (SD): 55.25 (9.86)
**Sex**	Female: 20 (100%)
**Race**	
White	16 (80%)
Black or African American	2 (10%)
Asian	2 (10%)
**Country of Origin**	
USA	17 (85%)
Sweden	1 (5%)
Thailand	1 (5%)
Ukraine	1 (5%)
**Education**	
Lower than Bachelor’s	1 (5%)
Bachelor’s	10 (50%)
Higher than Bachelor’s	6 (30%)
Unreported	3 (15%)
**Stage of Cancer**	
Stage I	11 (55%)
Stage II	6 (30%)
Stage III	3 (15%)
**Time Since Breast Cancer Diagnosis**	
<1 year	1 (5%)
1–3 years	9 (45%)
3–5 years	10 (50%)

**Table 2.** Macro BA and Macro F1 for Modality-Specific LSTM Models across Time Window Sizes (2–7 days). Best per modality in bold. 95% CIs shown beneath each value.

(A) Macro Balanced Accuracy (BA)						
Modality	2 days	3 days	4 days	5 days	6 days	7 days
Majority Baseline	0.50	0.50	0.50	0.50	0.50	0.50
Fitbit	**0.62** [0.58, 0.66]	0.60 [0.56, 0.64]	0.55 [0.52, 0.58]	0.54 [0.51, 0.56]	0.56 [0.52, 0.60]	0.56 [0.52, 0.60]
EMA	**0.64** [0.59, 0.69]	0.61 [0.57, 0.65]	0.60 [0.55, 0.64]	0.57 [0.53, 0.61]	0.56 [0.51, 0.61]	0.57 [0.52, 0.62]
MEMS	**0.73** [0.70, 0.77]	0.70 [0.65, 0.75]	0.71 [0.67, 0.75]	0.71 [0.67, 0.75]	0.70 [0.66, 0.75]	0.68 [0.63, 0.73]
(B) Macro F1						
Modality	2 days	3 days	4 days	5 days	6 days	7 days
Majority Baseline	0.50	0.50	0.50	0.50	0.50	0.50
Fitbit	**0.66** [0.61, 0.71]	0.63 [0.54, 0.74]	0.57 [0.44, 0.69]	0.59 [0.48, 0.71]	0.64 [0.55, 0.74]	0.64 [0.55, 0.74]
EMA	**0.66** [0.56, 0.76]	0.62 [0.52, 0.73]	0.61 [0.51, 0.72]	0.50 [0.40, 0.61]	0.62 [0.52, 0.71]	0.55 [0.44, 0.66]
MEMS	**0.84** [0.79, 0.89]	0.83 [0.77, 0.89]	0.83 [0.78, 0.88]	0.82 [0.76, 0.88]	0.81 [0.75, 0.87]	0.81 [0.75, 0.87]

Note. Majority baseline predicts the most frequent class; macro BA and macro F1 are both 0.50.

**Table 3.** Macro-Averaged Balanced Accuracy (BA, top) and F1-score (bottom) across modality combinations and time windows (2–7 days). Bold indicates the best within each combination. Values in brackets are 95% confidence intervals.

(A) Macro Balanced Accuracy (BA)						
Combination	2 days	3 days	4 days	5 days	6 days	7 days
Majority Baseline	0.50	0.50	0.50	0.50	0.50	0.50
EMA + Fitbit (Baseline)	0.62 [0.58, 0.67]	0.60 [0.55, 0.64]	0.58 [0.53, 0.62]	0.60 [0.57, 0.64]	0.58 [0.53, 0.62]	0.59 [0.55, 0.63]
EMA + Fitbit (Opt.)	**0.65** [0.60, 0.70]	0.62 [0.57, 0.67]	0.60 [0.56, 0.64]	0.60 [0.56, 0.63]	0.59 [0.54, 0.64]	0.56 [0.52, 0.60]
MEMS + EMA (Baseline)	0.72 [0.68, 0.76]	0.72 [0.68, 0.77]	0.67 [0.62, 0.72]	0.68 [0.63, 0.73]	0.70 [0.65, 0.76]	0.71 [0.65, 0.76]
MEMS + EMA (Opt.)	**0.73** [0.69, 0.77]	0.73 [0.69, 0.77]	0.72 [0.67, 0.77]	0.72 [0.67, 0.77]	0.73 [0.68, 0.78]	0.72 [0.68, 0.78]
MEMS + Fitbit (Baseline)	0.77 [0.73, 0.81]	0.70 [0.66, 0.74]	0.71 [0.67, 0.76]	0.65 [0.61, 0.69]	0.62 [0.57, 0.67]	0.61 [0.56, 0.66]
MEMS + Fitbit (Opt.)	**0.79** [0.74, 0.84]	0.69 [0.64, 0.74]	0.70 [0.65, 0.75]	0.64 [0.60, 0.68]	0.60 [0.55, 0.64]	0.63 [0.59, 0.67]
EMA + Fitbit + MEMS (Baseline)	0.81 [0.78, 0.84]	0.72 [0.68, 0.77]	0.66 [0.61, 0.70]	0.65 [0.60, 0.70]	0.63 [0.57, 0.68]	0.64 [0.59, 0.68]
EMA + Fitbit + MEMS (Opt.)	**0.83** [0.79, 0.88]	0.72 [0.68, 0.76]	0.70 [0.65, 0.75]	0.68 [0.63, 0.73]	0.61 [0.55, 0.66]	0.62 [0.57, 0.67]
EMA + Fitbit + MEMS + Baseline Survey (Baseline)	0.72 [0.67, 0.77]	0.67 [0.63, 0.71]	0.65 [0.60, 0.70]	0.62 [0.57, 0.66]	0.57 [0.52, 0.61]	0.58 [0.54, 0.62]
EMA + Fitbit + MEMS + Baseline Survey (Opt.)	**0.84** [0.80, 0.88]	0.71 [0.68, 0.75]	0.67 [0.63, 0.72]	0.68 [0.63, 0.73]	0.64 [0.58, 0.69]	0.60 [0.55, 0.64]
(B) Macro F1-score						
Combination	2 days	3 days	4 days	5 days	6 days	7 days
Majority Baseline	0.50	0.50	0.50	0.50	0.50	0.50
EMA + Fitbit (Baseline)	0.65 [0.56, 0.75]	0.65 [0.56, 0.75]	0.60 [0.50, 0.72]	0.62 [0.52, 0.72]	0.64 [0.55, 0.74]	0.59 [0.50, 0.68]
EMA + Fitbit (Opt.)	**0.68** [0.61, 0.76]	0.66 [0.57, 0.76]	0.64 [0.54, 0.75]	0.63 [0.52, 0.74]	0.64 [0.55, 0.74]	0.61 [0.52, 0.72]
MEMS + EMA (Baseline)	0.83 [0.78, 0.88]	0.83 [0.78, 0.88]	0.81 [0.76, 0.86]	0.81 [0.77, 0.86]	0.81 [0.76, 0.87]	0.79 [0.72, 0.86]
MEMS + EMA (Opt.)	**0.84** [0.79, 0.89]	0.84 [0.80, 0.89]	0.83 [0.77, 0.88]	0.82 [0.77, 0.88]	0.83 [0.78, 0.89]	0.81 [0.76, 0.87]
MEMS + Fitbit (Baseline)	0.84 [0.81, 0.88]	0.79 [0.72, 0.84]	0.81 [0.75, 0.86]	0.79 [0.73, 0.84]	0.79 [0.73, 0.84]	0.78 [0.72, 0.84]
MEMS + Fitbit (Opt.)	**0.88** [0.85, 0.91]	0.77 [0.70, 0.84]	0.81 [0.75, 0.87]	0.77 [0.73, 0.83]	0.76 [0.69, 0.82]	0.76 [0.70, 0.81]
EMA + Fitbit + MEMS (Baseline)	0.80 [0.74, 0.85]	0.86 [0.82, 0.89]	0.73 [0.66, 0.81]	0.73 [0.66, 0.80]	0.74 [0.68, 0.81]	0.76 [0.69, 0.82]
EMA + Fitbit + MEMS (Opt.)	**0.88** [0.84, 0.91]	0.82 [0.77, 0.87]	0.81 [0.75, 0.86]	0.73 [0.66, 0.80]	0.74 [0.67, 0.81]	0.74 [0.69, 0.79]
EMA + Fitbit + MEMS + Baseline Survey (Baseline)	0.85 [0.80, 0.90]	0.83 [0.78, 0.88]	0.79 [0.73, 0.86]	0.79 [0.74, 0.84]	0.79 [0.73, 0.84]	0.80 [0.74, 0.86]
EMA + Fitbit + MEMS + Baseline Survey (Opt.)	**0.88** [0.85, 0.92]	0.81 [0.75, 0.86]	0.78 [0.71, 0.84]	0.74 [0.66, 0.81]	0.82 [0.76, 0.87]	0.72 [0.66, 0.78]

Note. Majority baseline predicts the most frequent class; macro BA and macro F1 are 0.50 regardless of class imbalance.

“Opt.” = optimized soft-voting; “Baseline” = uniform weight voting.

**Table 4.** Comparison of Tier 1 Single-Modality Models and Tier 2 Optimized Fusion Models. Values in square brackets denote 95% confidence intervals.

Modality / Fusion	BA	F1-Score
Fitbit (Tier 1)	0.62 _[0.58, 0.66]_	0.66 _[0.61, 0.71]_
EMA (Tier 1)	0.64 _[0.59, 0.69]_	0.66 _[0.56, 0.76]_
MEMS (Tier 1)	0.73 _[0.70, 0.77]_	0.84 _[0.79, 0.89]_
EMA + Fitbit (Fusion, Optimized)	0.65 _[0.60, 0.70]_	0.68 _[0.61, 0.76]_
MEMS + EMA (Fusion, Optimized)	0.73 _[0.69, 0.77]_	0.84 _[0.79, 0.89]_
MEMS + Fitbit (Fusion, Optimized)	0.79 _[0.74, 0.84]_	0.88 _[0.85, 0.91]_
EMA + Fitbit + MEMS (Fusion, Optimized)	0.83 _[0.79, 0.88]_	0.88 _[0.84, 0.91]_
EMA + Fitbit + MEMS + Baseline Survey (Fusion, Optimized)	0.84 _[0.80, 0.88]_	0.88 _[0.85, 0.92]_

## Box

CCS Concepts: • **Applied computing** → **Health informatics**; Life and medical sciences; • **Computing methodologies** → *Machine learning approaches*; • **Human-centered computing** → Ubiquitous and mobile computing systems and tools.

## Footnotes

1
https://www.rxcap.com/

2
https://www.wisepill.com/

## A EMA Survey Instruments

This appendix summarizes the full set of Ecological Momentary Assessment (EMA) instruments administered during the study.

Morning Survey

- **Sleep** [43]
  – Hours of sleep last night (open-ended)
  – Sleep quality (1 = Very good to 5 = Very bad)
- **Pain and Fatigue** [29]
  – Pain level (0 = No pain to 10 = Worst pain imaginable)
  – Fatigue level (0 = No fatigue to 10 = Extremely severe fatigue)
- **Mood and Arousal** [12]
  – Happy, Sad, Calm, Sleepy, Anxious, Alert, Quiet, Irritated, Excited (1 = Not at all to 5 = Very much)

Evening Survey

- **Stress** [53]
  – Stressful event today? (0 = No, 1 = Yes)
  – If yes: Unpleasantness rating (1 = Not at all to 7 = Extremely)
- **Pain, Fatigue, Mood** (Same as Morning Survey)
- **Cognition** [53] (1 = Not at all to 5 = Very much)
  – How distracted were you today?
  – Trouble concentrating today?

Bi-weekly Survey

- **PHQ-4** [21] (0 = Not at all to 3 = Nearly every day)
  – Nervous/anxious, Uncontrollable worrying, Depressed/hopeless, Little interest
- **Fear of Cancer Recurrence** (0 = Not at all to 4 = A great deal)
  – Worry about recurrence
- **Medication Adherence** (0 = No, 1 = Yes)
  – Ran out of pills, Stopped taking, Forgot dose, Missed dose, Chose not to take
- **Symptoms** [40] (0 = No, 1 = Yes; severity if yes: 0–4)
  – Night sweats, Nausea, Bladder issues, Vaginal dryness, Pain with intercourse, Aches, Joint pain, Muscle stiffness, Concentration difficulty, Weight gain/loss, Appetite loss, Genital irritation, Diarrhea, Cold sweats

Monthly Survey

- **Healthcare Utilization**
  – Number of appointments, Types of providers seen
- **Physician Interaction Quality** [6](1 = Do not agree to 5 = Fully agree)
  – Interest in problems, Information quality, Trust, Shared decision-making, Empathy
- **Hospitalization and Surgery** (0 = No, 1 = Yes)
- **Social Support** (1 = Never to 5 = Always)
  – Emotional and instrumental support availability
- **Barriers to Care** (0 = No, 1 = Yes)
  – Transportation, Healthcare cost, Insurance, Prescription refills

Random Survey

- **Medication Context:**
  – Location during last medication intake
  – Activities during medication intake
  – Companions present
  – If not alone: Pleasantness rating (1 = Unpleasant to 7 = Pleasant)

## B Daily Fitbit Feature List

The following list enumerates all daily variables derived from Fitbit data streams and included in the modeling analyses.

**Steps** Total number of steps recorded per day.

**TotalDistance** Total distance traveled per day (in kilometers or miles).

**Calories** Total calories burned per day.

**LoggedActivitiesDistance** Distance logged during manually recorded activities.

**VeryActiveDistance** Distance during very active movement periods.

**ModeratelyActiveDistance** Distance during moderately active movement periods.

**LightActiveDistance** Distance during light activity periods.

**SedentaryActiveDistance** Distance accumulated during sedentary periods.

**VeryActiveMinutes** Minutes spent in very active intensity zones.

**FairlyActiveMinutes** Minutes spent in fairly active intensity zones.

**LightlyActiveMinutes** Minutes spent in light activity.

**Floors** Number of floors climbed.

**MarginalCalories** Estimated calories burned excluding basal metabolic rate.

**RestingHeartRate** Average resting heart rate (bpm).

**TotalMinutesWearTime** Total minutes the device was worn during the day.

**MinutesAsleep** Total minutes asleep.

**TotalTimeAwake** Total minutes awake during the sleep period.

**TotalMinutesLight** Minutes spent in light sleep stages.

**TotalMinutesDeep** Minutes spent in deep sleep stages.

**TimeInBed** Total time spent in bed.

**MinutesREM** Minutes spent in REM sleep stages.

**Sleep_Efficiency** Ratio of minutes asleep to time in bed (percentage).

**heartrate_mean** Mean heart rate during the day.

**heartrate_max** Maximum recorded heart rate during the day.

**heartrate_min** Minimum recorded heart rate during the day.

**heartrate_std** Standard deviation of daily heart rate.

**heartrate_skewness** Skewness of the daily heart rate distribution.

**heartrate_kurtosis** Kurtosis of the daily heart rate distribution.

**heartrate_variance_of_local_homogeneity** Variance of local homogeneity in the heart rate signal (measure of regularity).

**Day_Steps** Total number of steps during daytime hours.

**Night_Steps** Total number of steps during nighttime hours.

**Mean_Hourly_Steps** Mean steps per hour across the day.

**Step_Variability** Standard deviation of hourly step counts within the day.

**Num_Active_Bouts** Number of continuous activity bouts detected.

**Avg_Active_Bout_Length** Average duration (minutes) of active bouts.

**Max_Active_Bout_Length** Maximum duration (minutes) of an active bout.

**Num_Sedentary_Bouts** Number of sedentary bouts detected.

**Avg_Sedentary_Bout_Length** Average duration (minutes) of sedentary bouts.

**Max_Sedentary_Bout_Length** Maximum duration (minutes) of a sedentary bout.

**Day_Mean_HR** Mean heart rate during daytime.

**Night_Mean_HR** Mean heart rate during nighttime.

**ActiveSteps** Total number of steps during active periods.

**MaxSteps** Maximum number of steps recorded in a single minute.

**StepsVariance** Variance of steps across the day.

**StepsStdDev** Standard deviation of steps across the day.

**StepsSkewness** Skewness of the daily step distribution.

**StepsKurtosis** Kurtosis of the daily step distribution.

**LongestActivePeriod** Duration of the longest continuous active period.

**LongestSedentaryPeriod** Duration of the longest continuous sedentary period.

**ActiveBoutCount** Total number of active periods detected.

**SedentaryBoutCount** Total number of sedentary periods detected.

**CircadianAmplitude** Amplitude of the fitted 24-hour circadian rhythm model based on daily step count time series.

**CircadianFrequency** Estimated circadian rhythm frequency (cycles per 24 h) derived from step count rhythms.

**CosinorMesor** Mesor (mean level) from cosinor rhythm fitting of the step count series.

**CosinorAmplitude** Amplitude from cosinor fitting (strength of the step-based circadian rhythm).

**CosinorAcrophase** Acrophase (peak time) from cosinor fitting of the step-based circadian rhythm.

**HighIntensityDuration** Total duration of high-intensity activity periods (step rate above active threshold).

## C Baseline Survey Instruments

The following standardized instruments were included in the baseline assessments:

- PROMIS Global Health (PGH)
- Self-Administered Comorbidity Measure (SACM)
- Breast Cancer Prevention Trial Checklist (BCPTC)
- PROMIS Sleep Disturbance Short Form (SD)
- PROMIS Sleep-Related Impairment Short Form (SRI)
- Fatigue Symptom Inventory (FSI)
- PROMIS Pain Region (PR) and Pain Intensity 3a (PPI)
- Pain Catastrophizing Scale (PCS)
- Hot Flash-Related Daily Interference Scale (HFRDIS)
- Cognitive Function Short Form 8a (CFSF)
- Mini IPIP (MIPIP) Personality Inventory
- Pearlin Mastery Scale (PEARL)
- Functional Assessment of Chronic Illness Therapy-Spiritual Well-Being (FACIT-SP)
- Perceived Stress Scale (PSS)
- Generalized Anxiety Disorder Scale (GAD-7)
- Patient Health Questionnaire Depression Scale (PHQ-8)
- Fear of Cancer Recurrence Inventory (FCRI)
- Medication Adherence Self-Efficacy Scale (MASES)
- Medication Adherence Report Scale Items (MARSI)
- Beliefs about Medicines Questionnaire (BMQ)
- Perceptions of Endocrine Therapy Necessity (PETN)
- Decisional Regret Scale (DRS)
- Patient Satisfaction with Cancer Care (PSCC)
- Interpersonal Processes of Care Survey (IPCS)
- Patient-Perceived Self-Efficacy Scale (PPSE)
- Participation in Decision and Relationship Quality Scale (PDRQ)
- PROMIS Emotional Support (PES)
- PROMIS Instrumental Support (PIS)
- PROMIS Informational Support (PIFS)
- Barriers to Care Scale (BACS)
- Communication and Attitudinal Self-Efficacy (CASE)

## D Baseline Classical Models (LR and RF)

To contextualize the performance of our deep learning models, we compared them against two classical machine learning baselines: Logistic Regression (LR) and Random Forest (RF). These models were trained using only the previous day’s modality-specific features to predict the medication adherence status of the next day.

Table 5 summarizes the macro-averaged BA and macro-averaged F1 for each modality. Note that only Fitbit and EMA features were used in this comparison. We did not include MEMS as a baseline modality because MEMS data only provides binary adherence labels (i.e., whether the medication was taken), and it does not contain any predictive features beyond the previous adherence status itself. As such, MEMS is not a feature source but rather the prediction target in this study.

**Table 5.** Performance of classical baseline models (Logistic Regression and Random Forest) using only previous-day data to predict next-day adherence. MEMS is not included because it does not contain predictive features.

Modality	Model	BA	F1
Fitbit	Logistic Regression	0.56	0.76
Fitbit	Random Forest	**0.58**	0.72
EMA	Logistic Regression	**0.52**	0.67
EMA	Random Forest	0.47	**0.75**

## E Comparison of Deep Learning Architectures

To validate our choice of using LSTM networks as the primary modeling architecture, we additionally implemented two widely used sequential deep learning models: Transformer and TCN. These models were applied to the same modality-specific inputs (Fitbit, EMA, and MEMS) across varying input windows (2–7 days). For EMA, we also examined a reduced version containing only morning and evening surveys, excluding biweekly, monthly, and random entries, as suggested to reduce participant burden.

Table 6 presents the macro-averaged BA and macro-averaged F1, along with their corresponding 95% CIs, across all configurations. Overall, LSTM models consistently outperformed or matched the performance of Transformer and TCN models across most time windows and modalities. This performance gap was particularly pronounced in the MEMS and EMA settings, where LSTM demonstrated both higher average scores and narrower CIs, underscoring its robustness in modeling adherence-related temporal patterns.

Interestingly, the reduced EMA input (morning + evening only) led to only a modest drop in balanced accuracy compared to the full EMA configuration. For example, under the 2-day window with LSTM, the full EMA model achieved a macro-averaged BA of 0.64, while the reduced-input model achieved 0.62. This suggests that substantially reducing participant burden through fewer prompts may still retain acceptable predictive performance.

From a theoretical perspective, the superior performance of LSTM can be attributed to its inherent architectural properties. LSTM networks are specifically designed to capture short-term and medium-term temporal dependencies by leveraging gated memory cells, which regulate the flow of past information. This makes LSTMs particularly effective in modeling individual-level medication behavior patterns over consecutive days. In contrast, Transformer models, while powerful in capturing long-range dependencies, typically require larger datasets to fully leverage their self-attention mechanisms and may be more prone to overfitting on small and noisy health data. Similarly, while TCNs benefit from hierarchical receptive fields through dilated convolutions, they may underperform in tasks where fine-grained day-to-day variations—rather than multi-scale patterns—are most predictive of outcomes.

Taken together, these findings empirically and theoretically support our selection of LSTM as the primary modeling architecture for medication adherence prediction.

**Table 6.** Macro-averaged Balanced Accuracy (BA, top) and F1-score (F1, bottom) for modality-specific LSTM, Transformer, and TCN models across input window sizes (2–7 days). Best F1 per (model, modality) in bold. EMA^b^ reflects a reduced-input version of EMA. Values in brackets are 95% confidence intervals.

(A) Macro Balanced Accuracy (BA)							
Model	Modality	2 days	3 days	4 days	5 days	6 days	7 days
LSTM	Fitbit	**0.62** [0.58, 0.66]	0.60 [0.56, 0.64]	0.55 [0.52, 0.58]	0.54 [0.51, 0.56]	0.56 [0.52, 0.60]	0.56 [0.52, 0.60]
Transformer	Fitbit	**0.61** [0.58, 0.64]	0.59 [0.56, 0.73]	0.61 [0.58, 0.64]	0.58 [0.54, 0.62]	0.55 [0.53, 0.58]	0.56 [0.53, 0.60]
TCN	Fitbit	0.56 [0.53, 0.59]	**0.56** [0.53, 0.59]	0.54 [0.50, 0.58]	0.54 [0.51, 0.57]	0.54 [0.51, 0.57]	0.53 [0.49, 0.57]
LSTM	EMA	**0.64** [0.59, 0.69]	0.61 [0.57, 0.65]	0.60 [0.55, 0.64]	0.57 [0.53, 0.61]	0.56 [0.51, 0.61]	0.57 [0.52, 0.62]
LSTM	EMA^b^	0.62 [0.56, 0.68]	0.59 [0.54, 0.65]	**0.62** [0.57, 0.65]	0.62 [0.57, 0.67]	0.60 [0.56, 0.64]	0.61 [0.57, 0.66]
Transformer	EMA	**0.65** [0.60, 0.70]	0.58 [0.54, 0.62]	0.58 [0.54, 0.62]	0.58 [0.53, 0.63]	0.59 [0.55, 0.64]	0.56 [0.52, 0.60]
TCN	EMA	**0.63** [0.59, 0.68]	0.63 [0.58, 0.68]	0.60 [0.55, 0.64]	0.59 [0.54, 0.64]	0.55 [0.51, 0.60]	0.55 [0.51, 0.60]
LSTM	MEMS	**0.73** [0.70, 0.77]	0.70 [0.65, 0.75]	0.71 [0.67, 0.75]	0.71 [0.67, 0.75]	0.70 [0.66, 0.75]	0.68 [0.63, 0.73]
Transformer	MEMS	**0.68** [0.64, 0.72]	0.66 [0.61, 0.70]	0.63 [0.59, 0.67]	0.63 [0.58, 0.68]	0.62 [0.57, 0.67]	0.61 [0.57, 0.65]
TCN	MEMS	**0.71** [0.67, 0.76]	0.70 [0.66, 0.74]	0.72 [0.68, 0.76]	0.71 [0.66, 0.75]	0.71 [0.67, 0.75]	0.70 [0.66, 0.74]
(B) Macro F1-score							
Model	Modality	2 days	3 days	4 days	5 days	6 days	7 days
LSTM	Fitbit	**0.66** [0.61, 0.71]	0.63 [0.54, 0.74]	0.57 [0.44, 0.69]	0.59 [0.48, 0.71]	0.64 [0.55, 0.74]	0.64 [0.55, 0.74]
Transformer	Fitbit	**0.65** [0.58, 0.73]	0.57 [0.46, 0.68]	0.65 [0.56, 0.74]	0.60 [0.49, 0.71]	0.63 [0.53, 0.73]	0.59 [0.48, 0.70]
TCN	Fitbit	0.60 [0.51, 0.69]	**0.62** [0.52, 0.73]	0.65 [0.57, 0.74]	0.62 [0.53, 0.72]	0.62 [0.52, 0.72]	0.64 [0.55, 0.73]
LSTM	EMA	**0.66** [0.56, 0.76]	0.62 [0.52, 0.73]	0.61 [0.51, 0.72]	0.50 [0.40, 0.61]	0.62 [0.52, 0.71]	0.55 [0.44, 0.66]
LSTM	EMA^b^	0.62 [0.51, 0.73]	0.62 [0.51, 0.72]	**0.67** [0.59, 0.75]	0.67 [0.59, 0.75]	0.67 [0.59, 0.75]	0.62 [0.52, 0.73]
Transformer	EMA	**0.66** [0.55, 0.77]	0.66 [0.58, 0.74]	0.60 [0.51, 0.69]	0.60 [0.49, 0.70]	0.62 [0.53, 0.71]	0.60 [0.51, 0.70]
TCN	EMA	**0.70** [0.63, 0.78]	0.68 [0.60, 0.76]	0.62 [0.52, 0.71]	0.62 [0.53, 0.72]	0.58 [0.46, 0.70]	0.65 [0.55, 0.75]
LSTM	MEMS	**0.84** [0.79, 0.89]	0.83 [0.77, 0.89]	0.83 [0.78, 0.88]	0.82 [0.76, 0.88]	0.81 [0.75, 0.87]	0.81 [0.75, 0.87]
Transformer	MEMS	**0.77** [0.69, 0.85]	0.75 [0.67, 0.83]	0.74 [0.66, 0.82]	0.73 [0.63, 0.82]	0.73 [0.64, 0.82]	0.71 [0.60, 0.81]
TCN	MEMS	**0.83** [0.78, 0.88]	0.82 [0.77, 0.87]	0.81 [0.75, 0.87]	0.80 [0.74, 0.87]	0.81 [0.76, 0.87]	0.80 [0.74, 0.86]

*Notes*. Best F1 per *(model, modality)* in bold.

**EMA**^***b***^: Morning + Evening only (reduced-input EMA).

## F Age-based Subgroup Sensitivity

Participants were stratified by the cohort median age (50 years; <50 vs. ≥50), and LOPO evaluation was repeated using a 3-day input window. Performance metrics were computed per PID, macro-averaged, and 95% confidence intervals (CIs) obtained via PID-level bootstrap. Balanced accuracy (BA) was comparable across age groups for EMA and Fitbit models, while MEMS models performed strongly in both strata. Higher F1-scores in the ≥50 group were consistent with its higher adherence rate (Table 7), reflecting class-balance and operating-point effects rather than age-specific model failure. Overall, no systematic degradation in BA was observed for either age group.

**Table 7.** Sample sizes and adherence distribution by age subgroup (MEMS LSTM; 3-day input window).

Group	Number of Participants	Sequences	Adherent Days	Adherence Rate
Age<50	9	1481	850	0.57
Age≥50	9	1457	1222	0.84
All	18	2938	2072	0.71

Counts reflect MEMS-derived sequences used for the subgroup analysis; EMA/Fitbit sample sizes differ.

**Table 8.** Model performance by age subgroup (3-day input window; macro-averaged per PID with 95% CIs via bootstrap).

Modality	Metric	Age<50	Age≥50	All
EMA LSTM	BA	0.62 [0.57, 0.67]	0.59 [0.54, 0.65]	0.61 [0.57, 0.65]
	F1	0.63 [0.48, 0.76]	0.67 [0.49, 0.90]	0.62 [0.52, 0.75]
Fitbit LSTM	BA	0.64 [0.60, 0.69]	0.51 [0.48, 0.55]	0.60 [0.56, 0.64]
	F1	0.61 [0.54, 0.68]	0.78 [0.69, 0.87]	0.63 [0.54, 0.74]
MEMS LSTM	BA	0.72 [0.65, 0.78]	0.66 [0.59, 0.76]	0.70 [0.65, 0.75]
	F1	0.77 [0.67, 0.89]	0.78 [0.65, 0.94]	0.83 [0.77, 0.89]

## G Missingness Stress Test Analysis

To further evaluate the robustness of our framework, we conducted a sensitivity analysis using the time window = 3 configuration. In each held-out fold, we introduced synthetic missingness to simulate conditions beyond those observed in the real-world dataset. We evaluated two patterns of missingness: (i) missing completely at random (MCAR), where a fixed proportion of feature values (10%, 30%, or 50%) were independently masked across time steps; and (ii) structured gaps, where contiguous 3-day segments were masked in 10% of calendar weeks, approximating realistic scenarios such as multi-day device non-wear or missed survey submissions. All masked values were imputed with training-set feature means before normalization, representing a pragmatic deployment strategy in which missing inputs are replaced by population-level expectations.

As summarized in Table 9, predictive performance remained robust across all stress-test conditions. Balanced accuracy and F1 score declined by less than 0.02 on average relative to baseline models. Interestingly, EMA models showed small F1 improvements at higher MCAR rates, likely because random masking attenuated noise in self-reports. By contrast, Fitbit models were slightly more robust under structured gaps, consistent with their higher temporal density and redundancy. Together, these findings indicate that the proposed framework is resilient to moderate levels of additional data loss, whether random or structured, beyond the natural non-wear and skipped-survey patterns already present in practice.

## H Data Availability

At the time of publication, we are unable to publicly release the dataset used in this study due IRB restrictions. Researchers with specific questions about the dataset, analytic procedures, or reproducibility may contact the corresponding author to discuss potential options for data access under appropriate data use agreements and ethical approvals.

**Table 9.** Stress-test sensitivity analysis (time window = 3). Δ denotes change relative to the baseline performance of the same fold.

Modality	Condition	Rate	BA (Δ)	F1 (Δ)
EMA	MCAR	10%	0.60 (+0.01)	0.63 (+0.02)
EMA	MCAR	30%	0.59 (−0.00)	0.64 (+0.02)
EMA	MCAR	50%	0.58 (−0.01)	0.65 (+0.04)
EMA	3-day gaps (10% weeks)	–	0.58 (−0.01)	0.64 (+0.02)
Fitbit	MCAR	10%	0.60 (+0.00)	0.63 (+0.00)
Fitbit	MCAR	30%	0.60 (−0.00)	0.63 (+0.00)
Fitbit	MCAR	50%	0.58 (−0.02)	0.63 (+0.00)
Fitbit	3-day gaps (10% weeks)	–	0.59 (−0.01)	0.65 (+0.02)

We have released the complete source code and documentation replicating all preprocessing, modeling, and evaluation steps. The code is available at: https://github.com/BarnesLab/Endocrine-Therapy-Adherence-in-Breast-Cancer-Survivor.
